# Spirit of the Foreign Periodicals, &c.

**Published:** 1840

**Authors:** 


### Spirit of the Foreign Periodicals.

---

### Remarks on Stammering, by a Sufferer.

The following observations on this annoying infirmity are from the pen of a physician, who, till his twentieth year, had been afflicted with stammering, and who then cured himself in the manner which we shall afterwards notice.

Most writers on stammering have attributed it to the irregular and convulsive movements, or to the faulty position in relation to the teeth and palate, of the tongue and lips. Thus M. *Hervez de Chegoin* mentions the absolute or relative smallness of the flesh of the tongue, and the shortness of the frænum as its most frequent causes: and he has reported two cases where a cure was effected by dividing this bridle.

But in a large majority of stammerers there is no irregularity whatsoever in the organs of speech: and even if there was, the admission of such a cause would not account for the infinite varieties of the infirmity and the influence which moral emotions exert upon its development.

MM. *Sauvage* and *Itard* regard debility of the muscles of the tongue as one of the most frequent causes. But do not persons that stammer execute with the greatest ease and rapidity all the various movements of the tongue and lips?

Is it not rather a state of spasm than of debility? How too, on this hypothesis, are we to explain the spontaneous cure of stammering as life advances, when debility, local as well as general, must necessarily increase?

Other writers seek for the cause of the malady in the action of the brain on the muscles of the voice—an explanation which may be quite just, indeed, if it were not so vaguely expressed: a vagueness which seems to have actually bewildered some writers who have adopted the explanation in question. Thus, *Rullier*, in the Dictionnaire de Medecine, says:—" In stammering, the cerebral irradiation which follows an act of thought, and becomes the moving principle to set in action the muscles necessary to the oral expression of the ideas, bursts forth with such impetuosity, and is reproduced with such rapidity, that it exceeds the mobility of the agents of articulation."

But this is surely paying too great a compliment to stammerers; for my part I must confess that I never experienced that rapid flow of ideas and impetuous action of brain, which the author talks of.

M. *Magendie* attributes stammering to such a deficient action of the organic intelligence as is capable of disturbing the movements of the vocal organs, which are influenced at the same time by the nervous system of organic life, and by the action of the brain.

All that we can reply to this explanation is, to ask what the author means to announce; for to us his language is quite unintelligible.

But to form a more accurate idea as to the cause of stammering, let us now briefly notice what we observe in a person afflicted with this infirmity, when he attempts to speak.

In some cases—and this is the least severe form of the malady—the person speaks sufficiently fluently for a greater or less length of time; but if the pronunciation of a word which begins with certain consonants, such as *b*, *p*, or *v*, coincides with the close of an expiration, then the breathing becomes embarassed, hurried, and panting, and the efforts of the person to pronounce the word are accompanied with convulsive movements of the lips; at other times the lips are in a state of tonic spasm. At length the person succeeds in overcoming this difficulty, by taking his breath or making an inspiration.

In other cases, where the stammering is more decided, the person often cannot utter without difficulty almost any sound, even when the word which he wishes to pronounce begins with a vowel, (as was the case with myself in respect to the word *oui*); the features and neck swell up; the jugular veins become distended; and the person tosses his arms about to find relief.

These two degrees of the infirmity are often observed to co-exist in the same person: if he becomes embarassed by a word which begins with a consonant which is not easily pronounced, the fear of not being able to proceed renders the respiration panting; and then any efforts at speech are quite impracticable.

In both degrees or stages of the malady, *the stammering ceases after a strong inspiration;* but it returns at once if the person does not continue to respire regularly, and the more frequently and badly if his breathing becomes agitated, and he strives much against it.

*The great object, therefore, is to bring and keep the respiration in a quiet tranquil condition, and cause it to follow a certain degree of cadence, such as we use in singing or in declamation. If the person can but once effect this, he will no longer stammer.*

From what we have now said, it is evident that in all the various sorts or degrees of stammering, its essential cause consists in a convulsive condition, either of a tetanic or a choreic character, of the respiratory function—the effect of which is, either to disturb more or less, or altogether prevent, the articulation of sounds.

What occurs in the lips, tongue, and throat, is not the essential and primary cause, but is altogether accessory; and it is according to which of these parts is chiefly affected, that the different varieties of the infirmity are produced.

The immediate cessation of the irregular convulsive movements by taking in a deep inspiration, clearly shews how intimately they are connected with the cause now mentioned. It was by attending to this simple means, suggested to me by Dr. *Lindt* of Berne, that I cured myself at the age of twenty years. The cure, indeed, was aided a good deal, I believe, by the employment at the same time of gymnastic exercises—the effect of which is powerfully to increase the influence of the will over the muscular system in general. Indeed it has been by their influence in regulating the act of breathing, that the efficacy of various means, which have been recommended and found useful, at various times, in the treatment of stammering, is to be accounted for. In this way the practice of declaiming on the sea-shore with a pebble in his mouth cured, it is well known, Demosthenes of old.

M. *Itard* has advised the use of some mechanical means to keep the tongue raised, and he makes his patients at the same time speak in a foreign language. This plan of keeping the tongue raised towards the palate has been recommended by other writers. By this simple means, Madame *Leigh* assures us that she effected a cure in upwards of 150 cases.

But however useful it may be to a person who stammers to acquire a certain control over the movements of the tongue, let it be ever kept in mind, that no permanent benefit will be derived unless the patient acquires, at the same time, the power of regulating his breathing—so that whenever he begins to hesitate or falter, he at once draws in a full breath. Madame *Leigh* imposed upon her pupils absolute silence, except during the hours of practising: and she always observed that those who had the greatest mental energy and power over their will were the most easily and quickly cured.

M. *Colombat,* although his theory of stammering is far from being correct in our opinion, has recommended a most judicious method of treatment, which if rightly pursued is always sure to effect a cure. It consists in the adoption of the following three means: 1, giving the tongue such a position that its apex is directed upwards and backwards; 2, taking in a deep breath at the commencement of each phrase, and repeating this more or less frequently; and 3, marking the time in speaking by the movements of the thumb upon the fore finger.

By following out these simple rules, it is almost impossible for any one to stammer; and when we consider that this method of treatment consists essentially in causing the person to inspire at the commencement of each phrase, and marking the regular return of the inspirations by the movements of the thumb upon the finger, we at once understand its physiological explanation.

M. *Serres'* plan consists in making the person accompany the utterance of each sound with a powerful movement of the arms; he does not trouble himself about the situation of the tongue. Now this plan will often succeed as well as that which we have just recommended.

But neither M. *Serres* nor M. *Itard* his reporter, who is surprised at the singularity of this method, seem to have perceived that every muscular effort is in fact made during the act of expiration, and therefore that the pupil, to enable him to accompany the emission of any sound with a movement of his arms, must have always a provision of air in his lungs, and that thus he acquires the custom of speaking only during expiration.

There cannot be a question as to the utility of gymnastic exercises in increasing the energy of the will and the action of the brain upon the whole muscular system. It is a novel and a most practically useful idea of M. *Serres,* that the treatment of stammering should consist in a *gymnastique* of the respiratory and the vocal organs.—*Gazette Medicale.*

---

### Statistical Researches on Pneumonia.

The following conclusions are deduced from a careful comparison of 75 cases of active pneumonia observed in the wards of La Charité hospital, and under the care of M. *Bouillaud.* They have been collected by M. *Pelletan*; and his memoir, founded upon them, was recently reported upon to the Royal Academy by M. *Rayer* to whom it had been referred.

1. Pneumonia affecting one lung is more frequent than pneumonia of both lungs simultaneously;—in the ratio of 7 to 2.

2. The right lung is more frequently the seat of pneumonia than the left one —in the ratio of 2½ to 1. The greater frequency of the disease in the right side had already been noticed by previous writers, although they differ from M. *Pelletan,* and from each other as to the exact ratio of frequency.

3. The base of the lung is more frequently affected than the summit—in the ratio of 1½ to 1.

4. The age or duration of the pneumonia being the same, the degree or severity of the disease has been found to vary much in different cases; and it is only in the extreme limits that it has been possible to establish a relation between the duration of the inflammation and its development.

(The meaning of this sentence is anything but clear: we scarcely know what the author wishes to announce.)

5. The disease is twice as frequent between 17 and 37 years of age, as at every other period of life.

6. It is more frequent in men than in women;—in the ratio of 10 to 1.

7. The influence of cold as the exciting cause has been remarkable in seven-ninths of all the cases.

8. The frequency of the pulse has not afforded any exact measure or indication of the intensity of the disease nor of the point which it has reached. It is not, however, to be denied, that the acceleration of the pulse is a sign of considerable value, especially when it continues or increases after blood-letting.

9. The frequency of the inspirations has seemed to measure very exactly the degree of the disease, and to indicate its gravity.

10. Prostration and delirium have in general existed when the inflammation occupied the summit of the lung; this coincidence was of much more frequent occurrence than in pneumonia of the base.

11. As to the treatment of pneumonia, when it is *franchement inflammatoire,* repeated blood-letting, according to the formula of M. *Bouillaud,* formed its basis. There were only two deaths in 55 cases; and the duration of the disease was incontestably shortened.

12. Blisters were rarely useful in adults; occasionally in children; always in old persons.

M. *Rayer,* in reporting upon the memoir of M. *Pelletan,* very properly shewed that the question of the statistical or arithmetical history of pneumonia, or of any other disease, although apparently very simple if deduced from an ample number of cases, contains various elements which require to be attended to with the greatest care.

For in truth pneumonia is not the same malady in children as it is in adults and in old persons: and again, when it prevails epidemically, it is in many respects very different from that form which occurs in a plethoric person after exposure to cold. It is necessary also to take account of secondary pneumonias, of bilious complications, and of a multitude of other circumstances, before one can fairly draw any conclusions of general application.—*Gazette Medicale.*

*Remark.*—It will be observed by these very brief remarks of M. *Rayer* that he is a much less hasty reasoner, and less *heroic* practitioner than his confrere, M. *Bouillaud.—Rev.*

---

### SUGGESTION IN PARACENTESIS THORACIS.

*Case.*—A youth, after suffering for a month from a mucous fever, became affected with all the usual symptoms denoting a pleuritic effusion on the right side, which was fuller and more prominent than the other. Dr. *Petit* determined to perform paracentesis of the chest; but some hours before making the incision he cauterised the intercostal space with the potassa fusa. In the evening the cauterised part had become quite white, and its circumference was œdematous. After cautiously dividing the integuments and intercostal muscles with the scalpel, he reached the pleura, which he then opened. Immediately a stream of sero-purulent matter, very offensive, was discharged from the wound: altogether there must have been a pound. This patient gradually recovered, and ultimately he was restored to perfect health.

*Remarks.*—Dr. *Petit* considers that it is very useful in the operation for empyema to cauterise the spot where we intend to make an opening into the pleura. This has a double advantage:—1, the pain of the subsequent incision is very trifling; and, 2, the appearance of the integuments affords a very valuable diagnostic sign. For the facility with which the eschar becomes white, and with which the surrounding integuments are affected with œdema, will be found to indicate the presence of a subjacent fluid. This remark is applicable to the diagnosis of collections of fluid, simple or purulent, in all parts of the body.

Dr. *Petit* prefers the bistoury to the trochar in performing paracentesis thoracis. He very properly recommends that the condition of the liver and spleen be ascertained before we determine between what ribs the perforation should be made. He has known the application of the caustic on the intercostal space between the fifth and the sixth ribs fail of making an opening into the cavity of the pleura, and induce a peritonitis which proved fatal.—*Gazette Medicale.*

If M. *Petit's* observations are confirmed, they suggest a most useful hint in not a few cases, where a certain degree of uncertainty must always exist. —(*Rev.*)

---

### M. Pigeaux on the Diseases of the Heart.

It is almost unnecessary to allude to the interminable and ever-changing discordance of opinion of auscultatory writers on the seat and cause of the sounds of the heart.

M. *Pigeaux* dissents, as a matter of course, from the views of other writers, and has an opinion of his own, which by the bye he has materially modified from what it was a few years ago.

He describes the rhythm of the movements of the heart as embracing three periods—in the first the auricles contract, in the second there is a repose and silence; and in the last the ventricles contract. Here are his own words :—

"Let us suppose for an instant the heart entirely empty :—the blood then flows in upon it from all sides and in a continued stream, by the incessant emptying of the large veins, which terminate in it. The blood flows directly into the auricles, and as the auriculo-ventricular valves are down, it falls by its own weight into the ventricles until it fills them. When the heart is once full, let us see what takes place. The auricles will not contract first ; for such a movement could effect nothing, seeing that the ventricles are full at the time. The ventricles themselves therefore contract first : the blood pressed on all sides pushes back the auriculo-ventricular and the sigmoid valves ; these last give way and permit the blood to be propelled into the arteries........ Immediately after the contraction of the ventricles, the auricles contract in their turn, and project their contents into the ventricles, which at the time are partially empty and approximated to the ribs. After these two successive contractions follows the repose of the whole organ ; the heart falls into an entire in-action (resolution), which, it has generally been supposed, is simultaneous with the ventricular dilatation already completed. This repose lasts for a rather shorter period of time than that of the contraction. The ventricles finish by becoming filled during this interval, and the auricles continue their dilatation. Then, again, the rhythm of the movements recommences with the contraction of the ventricles ; the cardiac circulation is completed."

From this extract it appears that M. *Pigeaux,* has reproduced the opinion of M. *Laennec* himself as to the ryhthm of the heart's movements.

It may indeed be justly asked of him, what are the grounds for his resuscitating a theory which has been completely disproved and rejected by all recent writers ? What facts or experiments does he adduce to disprove the assertions of *Haller* and of many modern physiologists, among whom Dr. Hope deserves to be particularly distinguished, that the auricles contract before the ventricles, and that the contraction of these cavities follows immediately afterwards, without any intervening period of repose between these two movements ? M. *Pigeaux* has certainly not adduced any such facts in his work ; and he cannot therefore reasonably expect any one to believe it merely because such may now be his opinion ; and this opinion too differing very materially from *that* which he held a few years ago. Then he maintained that it was the shock of the blood propelled by the contraction of the auricles against the walls of the ventricles that was the cause of the first sound, and that the second sound was produced by the friction of the stream of blood on the parietes of the aorta when projected into this vessel by the ventricle.

In the following paragraph we have a summary of his present opinions:—

"*Explanation of the physiological sounds of the heart.*—1. First sound, contraction of the ventricles, dull sound, inferior sound caused by the friction of the blood against the walls of the ventricles, and the orifices and the parietes of the large blood-vessels.—2. Second sound, contraction of the auricles, clear sound, inferior or upper sound caused by the friction of the blood against the parietes of the auricles, the auriculo-ventricular orifices, and the cavity of the ventricles.—3. Period of silence and repose, the blood continues to distend the auricles and the ventricles, entering these cavities without producing any sound."

It has probably been to escape from the objection, fatal to his former opinion, that the first sound was not simultaneous with the diastole of the ventricle, that he has recently modified his views, and that he now ascribes this sound to the friction of the blood against the parietes of the ventricles during their contraction. The French reviewer of his work objects to his present doctrine in the following words:—

"We will not repeat our objections that the author has not supported his opinion with any direct facts or experiments, and that he has reversed the rhythm of the movements of the heart; but we will simply ask of him how he supposes that, when the ventricle is full, a sound can be caused by the friction of the particles of the blood against its parietes. During the contraction of the filled ventricle, the movement of the blood can take place only at the mouth of the aorta, and every other point of its parietes must remain quietly in contact with the liquid molecule which rests upon it; this molecule presses on the adjoining one, and this again on its neighbour; and thus the movement is communicated *de proche en proche* to those which are nearest to the arterial opening, where it (the movement) takes place. It is therefore only at the end of the ventricular contraction that the molecules in contact with the parietes of the cavity are set in motion;—whence it results that the first sound of the heart cannot originate in or commence with the friction of the blood-molecules against the ventricular parietes, and that, even at the close of the contraction, this friction must surely be too feeble to produce so distinct a sound as we hear on auscultation. We therefore cannot understand how, in the theory of M. *Pigeaux*, the first sound can be the lower or inferior one.

Neither can we admit the explanation given by M. *Pigeaux*; and although the objections which he has brought forward against the numerous theories which of late years have been proposed by different writers, are far from being destitute of force or foundation, we are obliged to choose from among those which are not directly contrary to the results of experiments, or in other words, which do not reverse the order of the movements of the heart."

It is indeed too true that there is still a most perplexing ambiguity as to the cause of the sounds of the heart, and that the wisest heads among the auscultators still differ very essentially from each other on this and some other points of cardiac pathology. How then shall he attach great value to those minute and finely drawn distinctions of auscultatory phenomena which some writers have attempted to establish? and even if these distinctions were really well founded, what practical good we ask would result from our knowing for example that it was the mitral or the tricuspid valve and not the sigmoid valves of the aorta or pulmonary artery that were affected in any particular case? The treatment in either case must be alike.—*Archives de Medicine.*

---

### ON THE AUSCULTATORY SIGNS OF THE EARLY PERIOD OF PHTHISIS.

M. *Fournet*, whose recently published Clinical Researches on the above subject have received the prize at the Concours of hospitals, dwells with great emphasis on the importance of attending to the *expiratory* as well as to the *inspiratory* sound during each act of respiration, as affording a most useful means of diagnosis in the early stage of pulmonary consumption.

"The respiratory murmur," he says, "if examined attentively with the ear, will be found to consist of two sounds, quite distinct the one from the other, and which correspond with the entrance of the air into the pulmonary cells and its expulsion from them. The double character of the murmur had not escaped the notice of *Laennec* himself; but it is chiefly to the observations of M. *Andral* and of Mr. *Jackson* (of Baltimore ?) that we are indebted for the first accurate remarks upon the practical advantages that may be derived from attending to it in the diagnosis of certain pulmonic diseases."

M. *Fournet* has very ingeniously laboured to shew that in a very early stage of the formation of tubercles in the lungs the inspiratory and the expiratory sounds are uniformly more or less altered from their normal characters, both as it respects their duration and their intensity, and this too at a period of the disease when auscultation, practised in the usual manner, affords none but the most negative results.

*The important alteration or modification consists in a diminution, more or less marked, of the duration and intensity of the inspiratory sound, and in a correlative and parallel augmentation of the duration and timbre of the expiratory sound.*

To use the numerical language employed by M. *Fournet* to denote the various degrees of change, the inspiratory sound in the healthy state being considered as 10 in reference to its duration and intensity, it may gradually decrease to 8, 6, 4, 2, or it may even cease almost entirely. The expiratory sound, on the other hand, becomes altered at the same time, but in an opposite sense; so that if we represent its healthy condition by the figure 2, it will be found gradually to rise to 4, 6, 8, 10, and even 20, until at length it becomes blended and confounded with the bronchial *souffle*.

This is precisely one of the principal results which *Jackson* and *Andral* had announced, before the appearance of M. *Fournet's* researches. Still great praise is due to him for having prosecuted the subject much further than his predecessors, and for having laid down its details with greater accuracy and precision than had been done before.

Once in possession of this fact, a fact which is supposed to indicate the presence of tubercles in the pulmonary parenchyma at a period anterior to that at which ordinary observation can detect it, M. *Fournet* proceeds to point out some abnormal sounds which accompany these modifications of the double respiratory murmur in phthisis. The most important of these are two sounds which seem to have hitherto escaped the attention of auscultators, and which our author designates the dry cracking and the dashing or rumpling pulmonary sounds (*bruits de craquement et de froissement.*)

(We deem it however unnecessary to follow our author in his description of these auscultatory phenomena, which after all are probably only modifications of the bronchial or the cavernous respiratory sounds. But as we have not yet perused his work, for ourselves we cannot say more.

On re-perusing some time ago the Lectures of Dr. Latham on Clinical Medicine—an admirable work of practical instruction on auscultation—we noted especially the following passages:—

"There may be, perhaps, now in the hospital a dozen patients who have cavernous respiration; and in each one of them the sound, besides being cavernous, has some distinct peculiarity; it is large, or it is small; it is a click, or a hum, or a squeak. It is like blowing into a bottle with a narrow neck, or into one with no neck at all; it is like the flapping of a valve; it is metallic; it is as if air was puffed *into* your ear, or as if air was drawn *from* it."

"The earliest and the least, but still a very authentic sign of vomica, derived from auscultation, is a mere click or slight ringing sound, heard in breathing, at some point beneath the clavicle or about the scapula, in a patient in whom all the surrounding parts have been for some time dull. This click, to remove all doubt of its being owing to the accidental lodgement of a piece of tough phlegm in one of the bronchi, must always be found at the same point on several examinations of the chest. It is one modification of cavernous respiration. It results from a cavity or vomica in its first formation, when the tuberculous matter is softening and when it is just beginning to admit air."—*Rev.*)

The following remarks on the treatment of the early stage of phthisis are worthy the notice of every reader.

"In judging of the nature of this disease, we must ever bear in mind that there are two elements which are invariably present; the one being general or constitutional, and consisting in an alteration of the nutritive function in consequence of an unhealthy state of the circulating fluids, and the other being local and consisting in a morbid degeneration of the pulmonary parenchyma. In conducting the treatment, therefore, we must never lose sight of this two-fold view. On the one hand we must employ such dietetic and medical means as are calculated to give tone to the whole organism, and render the blood more rich in fibrine; and on the other hand we are to endeavour to arrest the local morbid action, which is almost always of a sthenic character, by the use of appropriate topical remedies. It is in the judicious combination of these two classes of remedies that we can hope to found any rational method of treating pulmonary consumption. By fortifying the general system by means of a generous but not a stimulating diet, moderate and regular exercise in the open air, various forms of baths, &c., while at the same time we keep up a derivative action over the local seat of the malady by issues, blisters and such like means, we may reasonably hope to arrest the evil in not a few cases, which if improperly treated will rapidly hurry on to a fatal termination."—*Bulletin de Therapeutique.*

---

### APOPLEXY OF THE LUNGS, A FREQUENT CAUSE OF SUDDEN DEATH.

Taking the term *apoplexy* as designating an engorged or highly congested state of a part dependent on, or at least associated with, a partial paralysis of its nerves, it would indeed be rather surprising if the lungs were exempt from such a seizure.

. . . . . . . . "Why may we not presume that there may be a primary lesion of the functions of the pneumo-gastric nerves, inducing an extinction of the contractile and sensitive properties of the lungs, which will then become penetrated with serous or sanguineous fluid, just as the brain and its vascular apparatus are affected in similar circumstances: in truth, such cases are not unfrequent in old people, and in younger persons of a cachectic disposition. It is admitted by all that the encephalon is susceptible of a sudden compression, arising either from a mere turgescence of its vessels, or from a sanguineous effusion in consequence of the rupture of these vessels. In the same manner the substance of the lungs may become, as it were, inundated with an unusual quantity of blood, or this fluid may be extravasated from its vessels into the pulmonary tissue. Examples of both these conditions are not unfrequent in young persons who are of a vigorous and plethoric habit of body, especially after any violent exertion, as boxing, rowing, &c. Recovery from the former condition is not uncommon; the second form usually proves fatal.

*Bartholin,* in his Tractatus de Pulmone, states that, in many cases of sudden death, the cause is attributable to a sudden irruption of blood into the lungs, and a consequent stagnation of it in their soft texture.

The following remarkable instance is recorded by *Bonetus*, and entitled, *Suffocatio a crasso nigroque succo cordis sinus infarcirente ac posteriores lobos pulmonum.*

*Case.*—A man, 50 years of age, and of a robust habit of body, was commencing his breakfast, when his breathing became suddenly oppressed, and he felt as if he should be suffocated. In spite of the efforts of his physician he died about noon, retaining his faculties to the very last.

The body quickly became enormously emphysematous, and of a dark livid colour all over.

On dissection, the abdominal viscera were found in a healthy condition, with the exception of their appearing of a livid hue from a highly gorged state of their vessels. The texture of the lungs, especially in the posterior part of the lobes, was indurated and as black as if it had been soaked in ink. There was no fluid in the pericardium: the right cavities of the heart contained a dark-coloured blood, of a thick consistence but without coagula.

*Dionis* has recorded an interesting case of a similar nature:—

*Case* 2.—On the 16th July, 1691, the Marquis de Louvois, having dined *en bonne compagnie*, went to the council chamber to read a letter to his majesty. While reading he was obliged to stop, and left the palace immediately for his own house. He requested *Dionis* to bleed him immediately, as he felt as if he should be suffocated. His anguish became, nevertheless, still more distressing; and he complained of a peculiar sensation in his belly, as if it would open. In half an hour from the moment of seizure, he was a corpse.

On dissection, the brain was found to be quite normal; the stomach was full of food; the lungs were swollen and distended with blood; the heart was flabby, and its texture was of a remarkably soft consistence; not a drop of blood was in any of its cavities.

*Dionis* very properly attributed the death to a stoppage of the circulation; the lungs were filled with blood because it was retained in their parenchyma, and yet there was none in the heart, because none could flow from the lungs into its cavities.

(Most of our readers will doubtless regard this case of sudden death as the result rather of a cardiac than of a pulmonic lesion. The heart, it seems, was in a state of extreme *ramollissement*).

*Case* 3.—A gentleman, 54 years of age and of a strong habit of body, became affected with deep melancholy, in consequence of a sudden reverse of fortune; he had never suffered from any pulmonary distress.

He became, however, soon after this time subject to dyspnœa, and a sense of suffocation. The attacks of these symptoms were only transitory and occasional. While playing with some friends at *piquet*, he experienced a peculiar distressing feeling in the epigastric region, which obliged him to walk about his room. On sitting down he immediately expired

The body became quickly livid in different parts.

*Dissection.*—When an incision was made through the integuments of the throat, a quantity of blood flowed out; and, on pressing the abdomen, much frothy blood escaped from a wound in the trachea. On laying open this canal and the divisions of the bronchi, their lining mucous membrane was observed to be very highly injected. The pericardium was red and vascular on its inner surface, and contained a small portion of sanguinolent fluid. The aorta and pulmonary artery, as well as the entire surface of the heart, exhibited a deep red colour; the coronary veins were gorged with dark blood, but little was found within the auricles. The muscular tissue of the heart was much softened, and partially converted into a fatty or adipose substance: at one point there was found a mass of adipocire.

MM. *Legallois* and *Thillaye*, who examined the body, were of opinion that the immediate cause of death in this case was the effusion of blood in the bronchi, which caused a sudden suffocation. The passive dilatation of the left ventricle, which no doubt must have caused death sooner or later, could only have contributed to the fatal issue as far as it determined or favoured such an effusion.

*Case* 4.—The Duc de Fleury, 53 years of age, and of a highly nervous temperament, was recovering very favorably from a fracture of the leg when one evening, after drinking some almond emulsion, he was most unexpectedly seized with a feeling of extreme exhaustion; in half an hour afterwards he was a corpse. The impression was that he had been poisoned.

With the exception of an engorged state of the veins of the dura mater, and two points of ossification in the folds of the falx, no morbid appearances were discovered in the *encephalon*.

The *lungs* were so completely infiltrated with blood as to resemble a sponge which had been immersed in it. The heart was quite empty. On the stomach, and at different points of the intestines, there were patches of ecchymosis pervading the entire thickness of their parietes. The spleen was very large, of a soft consistence, and contained a quantity of dark fetid blood. The whole of the abdominal venous system was gorged and highly congested.

The preceding cases are sufficient to prove that many cases of sudden death are attributable to apoplexy not of the brain, but of the respiratory organs.

Let us now briefly consider what are the *causes* of such a lesion?

It is well known that, in examining the bodies of animals which have died from tying the nervi vagi, the lungs are always found gorged with blood, and the bronchi are obstructed with a reddish coloured sanies.

Now, as the ligature of these nerves necessarily induces a paralysis of the larynx and of the lungs, it seems highly probable that sudden death, when dependent upon pulmonary apoplexy, is also attributable to the same condition.

............ "The lungs, losing their contractility and sensibility, become passive, and permit the blood sent to them from the heart—which suffers but little from the interrupted functions of the nerves which it receives from each recurrent—to penetrate their texture; the power of the right ventricle becomes feebler as the lungs are more and more distended, and congested with blood; the glottis allows only a small quantity of air to pass at each act of inspiration, and thus the dyspnœa increases more and more until life is at length extinguished. Under such circumstances, the right cavities of the heart are found almost always much distended, while the left cavities are quite empty."

We may therefore fairly infer that paralysis of the nervi vagi is one, and perhaps the most frequent, of the causes of pulmonary apoplexy. Another cause is probably an increased force of the right ventricle of the heart, whereby a larger quantity of blood is sent, and this, too, with such power upon the lungs, that their tissue becomes oppressed and ultimately engorged.

If, at the same time, there be any obstruction to the return of the blood in the pulmonary veins into the left auricle, this engorgement must necessarily increase, and at length be so great as to give rise to extensive extravasation into the air-cells.

............ "The signs of the two forms of pulmonary apoplexy, to which we have alluded—viz. that dependent upon a paralysis of the nervi vagi, and that arising from the excessive impulsion of the blood through the pulmonic arteries—present certain differences which may serve to distinguish the one form of the disease from the other. The former is most frequent in advanced life, and at first is usually manifested by a sense of extreme weakness and of oppression; the patient grows pale, totters, and perhaps falls down. Nevertheless, he retains—and this circumstance alone is sufficient to shew that the attack is not of cerebral apoplexy—his intellect, and will tell the by-standers that all his distress is in the chest, and that unless relief is speedily given he must soon die. In perhaps a quarter or half an honr afterwards, a cold icy damp breaks out over all the surface, the pulse from being full and strong becomes rapidly weak and fluttering, and the mind begins to waver.

Most frequently the physician arrives only to witness the dying man, or perhaps his corpse; pronounces the case to be one of *apoplexie foudroyante,* and points to the brain as the seat of the attack

But how very different is the course of cerebral apoplexy! In it the patient at once, and from the very first moment, loses his intellect as well as sensation and power of motion, and he falls into a state of complete stupor; there is usually some distortion of the mouth, and more or less difficulty of swallowing, and the breathing is almost always slow and stertorous: this state usually lasts from twelve to twenty-four hours, or longer. Surely such a case as this has but few features in common with those exhibited by a sudden lesion of the nervous functions of the lungs."

Independently of the symptoms during life, our author is of opinion that the appearances presented by the corpse, before any dissection takes place, indicate the seat of the mischief.

"The eyes are open and projecting, as we notice in drowned persons; a frothy sanguineous mucus oozes from the mouth in greater or less quantity, as the head is elevated or not; and *the body retains for an unusual length of time its heat,* more especially in the epigastric region. Percussion of the chest gives out almost always a dull sound, and this can be attributed only to some recent lesion of the lungs, since, during the continuance of good health, no complaint had been made of the breathing. These details have nothing in common with those observed in the bodies of those who have died from cerebral apoplexy."

The danger of pulmonary apoplexy, especially in old age, seems to be much greater than that of apoplexy of the brain; life is more quickly extinguished, and remedial measures are altogether more inefficacious, And, indeed, we cannot be surprised at this, when we consider that if there be any paralysis of the nerves of the lungs, those of the heart must necessarily suffer at the same time; whereas in cerebral lesions, the movements of the heart are but little affected, in consequence of its chief nervous supplies being derived from the intercostals.

The experiments of *Le Gallois* have proved that the functions of the lungs will continue, as long as the nervi vagi are uninjured. While this, therefore, is the case, the blood continues to be arterialised, and to make its impression on the brain, as long as this organ is susceptible of receiving and transmitting it. But if the lungs be paralysed, the arterialisation of the blood ceases, and consquently all impression on the brain is soon suspended, and the nervous energy exhausted.

Before closing these remarks, we shall briefly notice two cases, to shew what we consider *threatened attacks* of pulmonary apoplexy in young persons.

*Case.*—A young girl, after violent exertion, was seized with shivering and other symptoms of fever. The breathing became much hurried and very short, and the face was swollen and deeply flushed. The patient was excessively restless, and occasionally expectorated blood; she found relief only by sitting close to an open window: there was also intense headache, dilatation of the pupils, obscurity of vision, short and rattling respiration, and the mouth was every now and then filled with a frothy sanguinolent saliva. The suffocation was imminent. The pulse was small, resisting and hard, *(serré)* : but, after a copious venesection which was practised, it became soft and compressible. The dyspnœa and tendency to stupor were also at once relieved; some leeches were then applied on each side of the neck, and next day the patient was nearly well.

*Case.*—A lady who had during her three first pregnancies suffered much from headache, vertigo, pains and sense of heat in the chest, attended with expectoration of blood, became pregnant for the fourth time. Again the same train of symptoms made their appearance ; she was bled ten times during her gestation, and all the period kept on a very light diet. By this treatment she went on to her full time, and she was safely delivered of a healthy child.

Dr. *Leveillé* embraces the substance of the preceding observations in the following three conclusions.

1. That apoplexy of the lungs has a perfect analogy with apoplexy of the brain.
2. That it (pulmonary apoplexy) almost always proves rapidly fatal, and generally more so than cerebral apoplexy.
3. That when death takes place in from a few minutes to one or two hours, the cause of it will be usually found in the chest and but seldom in the head.—*Revue Medicale.*

*Note.*—It is necessary to state that the memoir, from which the preceding observations are drawn, was read before the Institute of Paris so far back as the year 1815, and consequently before the development of the discoveries of recent pathologists on the influence of the diseases of the lungs, and more especially of the heart, in inducing sudden death. It is, however, not only interesting but really instructive occasionally to refer back to the opinions held by experienced physicians before the torch of auscultation was first applied by *Laennec* to illumine the obscurity which hung round thoracic pathology.

In perusing the preceding observations, the reader will probably remark that it is rather singular that the enlightened author should have so much overlooked the almost invariable coexistence of disease of the heart in cases of pulmonary apoplexy; although in almost every instance examined after death, this organ is described to have been flabby, attenuated, and loaded or even penetrated with fatty matter. The particulars indeed, as to the special lesion of its different cavities and their valves, are not given with sufficient minuteness to satisfy the modern pathologist; but we may fairly conclude that in the majority of the cases, at least in those which occurred in advanced life, the right ventricle was in a state of *ramollissement* and of dilatation. Now under such circumstances the impulsion of the blood along the pulmonary arteries is necessarily weak, irregular and uncertain ; hence there arises a congested state of the extreme vessels in different parts of the lungs ; and if at the same time there should appear to be any obstruction to the free return of the blood from the pulmonary veins into the left auricle, we cannot be surprised that the blood should escape, either by exudation or by extravasation from rupture into the loose cellular substance, and also, into the air-cells of the lungs, and thus give rise to the disease which has been designated *pulmonary apoplexy.*

Such being the explanation of the mischief, we can at once understand *how* it proves so quickly and often so inevitably fatal.

Not a few cases of asthma, or rather of permanent dyspnœa, in old people, are attributable to this state of things. There is no paralysis of the pulmonary nerves, as imagined by Dr. *Leveillé,* and no primary disease of the lungs themselves, further than their capillary vessels being more or less congested and therefore weakened : it is not so much a *vital* as a *mechanical* lesion.

Such being the fact, the judicious practitioner will, in the treatment of a case of what he may deem to be one of pulmonary apoplexy, avoid any active or very officious treatment, as the powers of life are so languid that any further depression must endanger the feeble spark of life that remains. On the whole perhaps, the best practice will be found to be with the one hand to relieve the local congestion by the application of numerous leeches or of the cupping instruments to the chest or back, and with the other hand, and at the same time, to administer frequent doses of ammonia and such diffusible stimuli. Blood-letting from the arm is in many cases inadmissible, at least until some degree of reaction from the primary collapse has taken place: the quantity drawn must depend upon the effects produced upon the general circulation; hence the physician must watch these most attentively while the blood is flowing.

The auscultation of the heart will be found to afford a safer and surer test to guide our practice than the examination of the arterial pulse. The exhibition at the same time of small doses of stimulants, more especially of ammonia and æther, or of hot brandy and water, is not at all inconsistent with moderate depletion. For the powers of the system must be kept up to enable us with safety to relieve the engorged vessels; and the very nicety of the management of such cases consists in the contemporaneous use of stimulating and of depletory measures.

As a matter of course these remarks are applicable chiefly to those cases of pulmonary apoplexy which occur in old people, and are accompanied with, if not absolutely dependent on, a weakened state of the right cavities of the heart. That form of the disease which is apt to occur in young plethoric persons, especially during severe and protracted labour or suffeiing, as for example during accouchement, requires a much more vigorous and more decided lowering treatment; a large opening must be made in a vein, and the blood be allowed to flow freely and copiously; repeated doses of tartar emetic at the same time, so as to induce nausea, will likewise be most useful.—(*Rev.*)

---

### M. Piorry on Hæmitis.

The term *hemite* has been given to that alteration of the blood which exists in inflammatory diseases, and which is characterised by the formation of the buffy coat on its surface when it has been drawn and allowed to rest. This condition of the blood has been recognised from the earliest period; but it has only been within the last few years that the constitutional disturbance occasioned by it, before any decided local inflammation has been set up, has been attended to as a special disease. The "inflammatory fever," and "Synocha," of older authors, the "Angeiotenic fever" of *Pinel*, and the "Rheumatic fever," of *Sarcone*, exhibit, it must be admitted, most of the symptoms which exist in genuine "hæmitis;" but the latter term is greatly preferable as being much more explicit, and as expressive of the pathological cause of the disease.

It is but fair to acknowledge that *Sydenham, Van Swieten, Boerhaave* and many other excellent physicians of the old school, seem to have been much better acquainted with this malady than the authors of more recent times: it is well known how constantly allusion is made in their writings to the over-plasticity of the blood causing obstructions in the smaller vessels, and thus giving rise to inflammation and its consequences—a doctrine which *Magendie* and some of the German writers of the present day have been endeavouring to re-establish.

After alluding to the influence of the size, depth, &c. of the vessel, into which the blood is received, on the production of the buffy coat—which, by the bye, has been much exaggerated by many—M. *Piorry* goes on to state :—" In 1826

I published an acconnt of numerous experiments to prove that *the serum of the blood contains the materials of the buffy coat, and that this is formed by precipitation.* More recently it has been found by microscopical examination, that it (the buffy coat) is composed of colourless globules of an albuminous or fibrinous nature."

It has been objected to M. *Piorry's* views on hæmitis, that the blood is not 'an organ,' and cannot therefore be susceptible of inflammation; to this he replies, "Bordeu has triumphantly answered this objection. In treating of the medical analysis of the blood, which he calls fluid flesh (chair coulante) he views it as so completely a living organ that he rejects all the analyses of mere chemists, and asserts that it is a physician only that can examine the blood properly. And is it not the case that, in every inflamed part, we must take into account alike the fluids and the solids, in other words alike the blood-vessels and the blood itself?"

M. *Piorry* proceeds to describe the appearances exhibited by the blood, in cases of hæmitis, during coagulation:—

"The two elements of the blood separate, the coagulum falling to the bottom and the serum resting uppermost; in other cases this is reversed: the separation takes place usually eight or ten minutes after the blood has been drawn from the vein. The serosity is not at first clear and transparent, but it is of a greyish opaline or pearly white colour; sometimes it is yellowish. The surface of the coagulum is at first uniformly red, but this soon begins to exhibit a greyish stratum, which is gradually deposited more and more as the serum becomes clearer, and loses its opaque opaline appearance. The buffy coat is thus formed because the serum is uppermost and floats above the serum."...... "The buffy coat adheres to the coagulum; its thickness varies; its colour is yellowish, greyish, and sometimes has a slightly green hue; occasionally it is mixed with some blood globules. Its consistence varies exceedingly in different cases; it is always the greatest at the surface: the supernatant serum is generally clear, transparent, and of a citrine colour."...... "If we remove with a syphon the serum from inflamed blood, as it separates from the coagulum, and put it on a serous membrane or indeed upon a glass, a deposit similar to the *couenne* or buffy coat will be observed to take place. This deposit is sometimes in a mass; at other times it separates into two portions, of which one falls to the bottom and the other mounts to the surface. If it be exposed to the air, it reddens(?) and resembles a good deal the fibrine precipitated from the blood when this is briskly stirred about with a cane upon being drawn from a vein."...... "The examination of the buffy coat with the microscope and with chemical means has shewn that the plastic lymph is formed from the serosity of the blood, and is composed of globules."

*Necroscopic appearances.*—" Most of the physical appearances exhibited by the blood after death are the same as what we have mentioned are observed in it when drawn from the veins during life; this fluid is more or less extensively coagulated in the heart and blood-vessels. *Laennec, Legroux,* and *Bouillaud* have described with much care the appearance of the coagula often found in the cavities of the heart and of the larger veins; occasionally too in one of the larger arteries. Another necroscopic sign of hæmitis is the occurrence of a layer of plastic lymph, very similar to the buffy coat of the blood, on the surface of some of the serous membranes, as the pleura, pericardium, peritoneum, and the synovial capsules. Sometimes also a blistered surface is found covered with a layer of dense coagulable lymph: and occasionally also we observe in the cellular tissue around any inflamed part a dense resisting layer of this lymph, which has been mistaken for a scirrhous formation.

Besides these signs, the lining membrane of the heart and blood-vessels often exhibits a most distinct vascular congestion."

*Cause.*—"In nine-tenths of the cases of hæmitis, the disease may be traced to the influence of cold, especially where the surface has been perspiring. Every one knows how almost invariably Pneumonitis, and Hæmoarthritis are attributable to this cause. Now let us remember that an inflamed state of the blood in a vast majority of cases precedes the invasion of the local symptoms, and is indicated by a feeling of general *malaise,* tendency to shivering, weariness and pains in the limbs and back, &c."

M. *Piorry* doubts that excesses in eating are apt, as supposed by many people, to induce hæmitis; they have a tendency, says he, to cause rather Polyhyperhæmia than hæmitis. Even the inordinate use of wine and spirituous drinks seldom seems to bring on this condition of the blood, unless the agency of cold be exerted on the body at the same time.

Disturbance of the respiratory function appears often to have a tendency to bring on an inflamed state of the circulating fluid; hence perhaps the frequency of fibrinous concretions in the heart in cases of Anhæmatosis.

*Symptomatology,*—It is difficult to describe the symptoms of hæmitis, apart from those of its complications—in other words, of the local inflammation which is almost invariably very quickly induced by it. One of its earliest symptoms is a shivering, which is generally more or less severe and lengthened in proportion as the disease is more or less decided; this is succeeded by a heat over the whole surface; the pulse becomes much quickened, the face is flushed, and the capillary vessels in every part seem to be highly injected. But it is unnecessary to pursue this part of the subject, as the description given by M. *Piorry* corresponds exactly with the symptoms of synocha or inflammatory fever of other authors. That he really regards these diseases as perfectly analogous is evident from what he says as to the rapid course of the slighter cases of *hæmitis:*

"If the hæmitis be slight, and if it will terminate favourably after a few days rest, a gentle diarrhœa comes, or the patient discharges a quantity of sedimentary urine, and the health is gradually restored:" and again, "at the commencement of all exanthematous fevers, during the whole period which precedes the appearance of the eruption, the symptoms of hæmitis are present, and yet there is no buffy state of the blood present."

Now for a few words as to the treatment recommended by M. *Piorry* in hæmitis. *Blood-letting* as a matter of course he enjoins. *Purgatives* he does not approve of; "they not only withdraw the serosity of the blood, but they irritate the intestine; a few glasses of Seidlitz water, or an enema occasionally are best." (How practical!) *Blisters* are decidedly useful, but not at the commencement of hæmitis; it is towards the close of the attack, and especially when there is Hydrohæmia or Anæmia that they are most beneficial. One of the most efficacious remedies in hæmitis is the copious use of *diluents:* "we can best diminish the plasticity of the blood, by making the patient drink very freely of any mild fluid; the blood becomes thus more and more diluted, and less disposed to coagulate."* To facilitate the absorption of the fluid into the blood, our author recommends at the same time the repeated administration of emollient enemata. (!) M. *Piorry* has no faith in any of the antiphlogistic remedies which have been supposed to counteract the over-plasticity of the blood:—"We have tried to prevent the formation of the *couenne* by the administration of

---

* M. *Piorry* remarks that if "we were to be guided by the results of mixing water with the serum of inflammatory blood out of the body, the employment of large quantities of diluents in hæmitis might be deemed any thing but prudent; for if we add pure water to the serum, there is a sudden coagulation induced. "But we must remember," he adds, "that diluents when swallowed reach the blood only after having been sufficiently elaborated."

the alkaline carbonates, the preparations of antimony, and various other chemical salts; none of these remedies has been of much advantage; we must receive with extreme reserve many of the statements of *Rasori* and his followers as to the efficacy of antimonials in the treatment of phlogistic diseases."—*Gazette des Medecins-Praticiens.*

*Remarks.*—Much of what M. *Piorry* has written on hæmitis, or the inflammatory condition of the blood, is strictly true; although unfortunately he has, like most authors on their peculiar or favourite subject, carried his ideas much beyond what is warranted by experience. That the blood is apt under certain circumstances, and more especially in certain habits of body, to acquire an abnormal degree of plasticity, or in other words to contain an unusual quantity of fibrinous or coagulable matter, cannot be disputed by any one: it is a fact of daily occurrence; and it is equally certain that this over-plastic state of the blood is a very general *precursor* of all decidedly inflammatory diseases. It is a common mistake to suppose that the invasion of these diseases is often quite sudden and is not preceded by any appreciable symptoms. We believe that in almost every case of decided phlegmasia the blood has been for several days, if not weeks, previous to the explosion (so to speak) of the local mischief, in a more or less altered state—a state corresponding with what M. *Piorry* has designated hæmitis. Many a severe attack might be, no doubt, prevented if this precursory state was detected sufficiently early, and means were used to impoverish and attenuate the state of the circulating fluids. We cannot indeed suppose that the blood can become so quickly charged with an excess of fibrine, as the suddenness of many an inflammatory attack may lead us to imagine: the train had been laid for some time before the explosion took place.

This inflammatory state of the blood is indicated by the following symptoms: general oppression and weariness after exertion and eating, occasional headache and dullness of the intellect, slight confusion of sight and perhaps tinnitus aurium, occasional nausea and sickness, scanty secretion of the urine which is almost always deep coloured and hot, flying pains in the joints and limbs, heavy and disturbed sleep, &c. Now this state of things may continue for weeks and even months before any local or well defined attack appear, and all the time the person may have been able to be going about and attending to his affairs, as usual. Suddenly, as is imagined, he is seized generally after exposure to damp and cold with shivering, followed by heat, and then the symptoms either of pneumonia, or acute rheumatism, pericarditis, &c. set in.

But if the truth were known, the attack has been by no means sudden; many a warning had been given to the patient that there was something not altogether right with him, and he in all probability had been trying to shake off his unpleasant symptoms not by lowering his diet, but rather by taking an additional quantity of stimulus in the form of wine, porter, &c. In this manner the *hæmitic* state of the circulating fluid gradually establishes itself, undiscovered alike by the patient, and probably also by his medical attendant. But we cannot at present extend these remarks. Suffice it therefore to say, that although we cannot agree with M. *Piorry* that every case of synocha is dependent upon hæmitis, we very willingly admit the practical value to be derived from a greater attention to the state of the blood as the groundwork or precursory stage of many inflammatory diseases; more especially of acute rheumatism, pneumonia, and pericarditis.

The first of these diseases—acute general rheumatism or rheumatic fever, as it is often called—is perhaps of all the one which is most essentially connected with that state of blood in which there is a great excess of the fibrinous portion; and it is more than probable that it is from this circumstance that we are to account for the very intimate dependence of pericarditis and many lesions of the heart and great blood-vessels on previous attacks of acute rheumatism. We thus more and more distinctly perceive the high importance of paying extreme attention to the cardiac symptoms during and after (not for weeks only but for months and years) an attack of severe rheumatism.

One word before we close, as to the treatment of *hæmitis;* for it is in this branch of his theme that the observations of M. *Piorry,* as indeed is the case with most of the medical writings of his countrymen, are most faulty. For example, he does not even once allude to the undisputed effects of mercury upon the over-fibrinous state of the blood; and yet we believe that most English practitioners would rather dispense with the lancet itself than with this most potent remedy in the treatment of hæmitis. Iodine and its preparations exert a similar effect. How far colchicum has any direct influence on the condition of the blood, we cannot say; but it seems probable that it has.

Once more, we strongly urge upon our readers' attention the importance of keeping an eye for a length of time upon such of their patients as have suffered from those inflammatory diseases in which the blood has been highly charged with fibrine; as the foundation of many organic diseases, especially in the thoracic viscera, is often imperceptibly laid during the following six or twelve months.—*Rev.*

---

### Case of Puzzling Abdominal Tumour, with Remarks.

The following case, which puzzled many of the leading men in Paris, is worthy of record, as a fair instance of the difficulty of forming a correct diagnosis in certain cases of abdominal enlargement in females. As the patient, a girl, ultimately recovered, it is still a matter of doubt what her disease really was. By some the swelling was attributed to *pregnancy,* by others (M. *Roux*) to *extra-uterine pregnancy,* by M. *Blandin* to an *encysted tumour of the ovary,* by M. *Montain* to the *presence of a dead and decomposed fœtus,* by M. *Recamier* to an *accumulation of fæculent matters in the colon,* by M. *Jobert* to an *extravasation of blood in the omentum or mesentery,* and lastly, by others to an *accumulation of blood in the cavity of the uterus.*

Let us now mark the particulars as related by M. *Boinet,* who seems to have watched the case during the whole of its lengthened duration.

In 1836, the patient, 16 years of age, was admitted into the Hôtel Dieu for retention of urine, accompanied with considerable tumefaction of the abdomen. The catamenia had appeared when she was only twelve years old, but they had been irregular in their re-appearance; and during the intervals there was a copious leucorrhœa. Her health had however continued very good till about a twelvemonth ago, when she began to suffer every now and then from nausea, indigestion, and palpitations of the heart. At this time she was admitted into the Hospital Beaujon, where she remained for ten months for a *pretended* chronic gastritis: the catamenia were absent for five months at a time. She complained of a dull and deep-seated pain on the left side of the abdomen—for several days it was sharp and darting; then it would subside; and again return. It was encreased on pressure; and the abdomen began to be much more swollen and hard than it had ever been before. For two months she was affected with a diarrhœa, which resisted the effects of every remedy. When this ceased, it was replaced by a retention of urine of a rather singular character. In a bath she could pass urine freely; but out of it not a drop would come except with the catheter. She was bled from the arm and with leeches, blistered, cupped, took steel, quinine, and various other remedies; but nothing that was tried seemed to have any effect in diminishing the enlargement of the abdomen and the uneasy tenderness on its left side.

She left the hospital and entered the Hôtel Dieu.

At this period her general health seemed to be perfectly good, if we might judge from the freshness of her complexion, and the plumpness of her whole body. Her appetite was regular and good, and her bowels acted daily. The action of the heart was strong and rather tumultuous; but, as her temperament was highly nervous and impressionable, the palpitations to which she was frequently subject, especially after any exertion, such as going up stairs or walking quickly, were deemed to be hysterical. To void urine she was still obliged to use a catheter, except when she was in a bath; then she was always able to pass it without difficulty.

The abdomen was excessively tumefied over its whole extent, so much so that it gave the spectator the idea of pregnancy at its full term. It was not painful, except when pressure was made between the pubes and umbilicus—but firm and resisting, although no traces of a distinct tumor could be felt anywhere.

On percussion, the right side of the abdomen was remarkably resonant, whereas the left, at least over the iliac region, was dull. Aucultation afforded no signs of any import.

The mammæ were small as in a virgin, and not at all painful: neither was there any discolouration of the areolæ.*

Examination per rectum and per vaginam, as well as of the bladder with the catheter, afforded no signs of the existence of any tumor in the pelvis.

As she suffered much from headache, and the catamenia were still absent, she was bled from the arm, and also from the feet, and had leeches on the thighs and on the pudenda. Various emmenagogues were also given, but without producing the desired effect.

M. *Blandin*, under whose care she was, and who was inclined to regard the case as one of encysted tumor, (probably ovarian,) requested the advice of several of his colleagues.

M. *Roux* thought it probable that there was extra-uterine pregnancy; and M. *Recamier*, that all the symptoms were probably attributable to an accumulation of fæces in the sigmoid flexure of the colon.

Acting upon this idea, M. *Blandin* put his patient upon a course of purgative medicines, and of cold bathing. But under this treatment the abdominal fullness increased rather than diminished, and the tenderness in the left flank became greater than it had been for some time.

From the Hôtel Dieu, she passed into the Hospital St. Louis, and put herself under the care of M. *Jobert*: she still retained all the appearance of plump health; but there was no change in the size of the abdomen, and the dysuria was as distressing as ever. While in the hospital, the girl said that she was sensible of water being in her abdomen; and although no fluctuation could be perceived by tapping with the fingers, a sound as if of a wave of fluid was heard, when the body was shaken briskly from side to side. The fluid seemed to be contained within a limited space; and this was exactly in the left iliac region, where percussion elicited a dull sound and a sense of pain and uneasiness was experienced on firm pressure. Still no distinct or circumscribed tumor was perceptible here or elsewhere, although the left side of the abdomen was now sensibly more distended than the right: the general health continued to be ex-

---

* Not satisfied with these and other signs sufficiently negative of pregnancy, the French physicians, with that disregard of delicacy which we fear is but too common on the continent, proceeded to examine, with all possible minuteness, the organs of generation, although the girl assured them of her chastity; and there is given a very particular account of their appearance, which we, however, have no intention of transcribing. Suffice it to say that every thing indicated that she was *une jeune fille sage*.

cellent. The girl had been taking the ergot of rye and steel pills for some time, when diarrhœa came on, and the catamenia made their appearance for a few days. This had the effect of diminishing somewhat the abdominal fullness: the excretion of the urine was, however, still as difficult as ever, and could be effected only while the patient was in a bath, unless a catheter was used.

Next month menstruation returned, although still scantily: the abdomen was becoming gradually somewhat smaller; the other symptoms were as before.

At the following period, the catamenia were much more abundant; the abdomen became now more tender, and sharp darting pains were experienced through it ever since the menses made their appearance; it was sonorous on percussion everywhere except in the left iliac region.

From this period menstruation was regular, the swelling became gradually less and less, and the tenderness on the left side diminished at the same time, so that at length the abdomen acquired its normal dimension, and no inconvenience remained, except a slight uneasiness over the former seat of suffering when firm pressure was made upon it.

The retention of urine continued for a considerable period; but this symptom also at last ceased spontaneously.

Just before each menstrual period, the patient continued to experience a rather sharp pain in the left groin; but this ceased when the catamenia made their appearance.

Such is the history of this very curious and instructive case. It is still a problem what its real nature was and to what cause the abdominal swelling was attributable.

The most probable conjecture seems to us to be, that there was a serous cyst connected with the left ovary, or with the left Fallopian tube, and that the contents of it were either absorbed or were discharged, perhaps, by the bowels.*

The following case gives some grounds of probability for this opinion.

*Case.*—A lady, 28 years of age, was excessively chagrined and desponding

---

* It will be observed, that one among the many conflicting opinions on this case was, that there was a retention of the catamenia in the cavity of the uterus. That such, however, was not the case, was at once decided by introducing a probe or catheter into the cavity of the uterus to the extent of an inch and a half—*a simple and conclusive test of the existence, or not, of foreign matters within the uterus.*

As to the idea that there was a genuine encysted drospsy of the ovarium, Dr. *Boinet* remarks:—

"When encysted dropsy is so far advanced as to cause a regular, uniform, and symmetrical tumefaction of the abdomen, there usually exist symptoms—such as the sense of fluctuation, functional disturbance of the viscera, distress in breathing, œdema of the lower extremities, development of the sub-cutaneous abdominal veins, &c.—which were not present in our patient.

The examination *per vaginam*, also, was far from confirming the idea that there was a dropsical enlargement of the ovarium; for, when this is the case, we usually find that the position of the uterus is more or less deranged, the fundus being drawn over to one side by the tumor, and its mouth and neck being inclined over to the opposite side; hence, the edges of the *os tincæ*, instead of being parallel, as in health, to the sacro-ischiatic symphyses, become the one superior and the other inferior, so that one of the surfaces corresponds with the sacro-vertebral symphysis, and the other with the anterior parietes of the hypogastrium."

after the sudden death of her first and only child. Although her health suffered a good deal in consequence, the catamenia returned regularly. On one occasion they ceased for a week or so, and, as her great desire was to have a family, she fondly hoped that she might be pregnant. But such was not the case; and, after the lapse of several months, she became more depressed than ever. About this time she began to experience a pain in the left side of the abdomen between the false ribs and the spine of the os ilii, and in this place a circumscribed fullness or tumor was distinctly perceptible: it was of a globular shape, not adherent to the abdominal parietes, moveable to a certain extent, and seemingly deep-seated, and of about the size of two fists joined together. What was the nature of this tumor? It might be an extra-uterine conception, or an encysted or hydatidic tumor of the ovary, or an accumulation of hardened fæces.

After the lapse of a month, all uncertainty was dissipated by the discharge of numerous hydatids with the stools, and the almost complete subsidence of the tumor. The lady became subsequently pregnant and gave birth to a child. —*Gazette Medicale*

---

### M. ANDRAL ON THE VALUE OF CHEMICAL AND PHYSIOLOGICAL EXPERIMENTS.

.......... "The more complex that the composition of any animal fluid is, the more uncertain are the results of its analysis.

Take, for example, the blood: what do we really know about it? Little more, indeed, than that it is a fluid which holds in suspension numerous globules of a determinate size, shape, and colour.

So difficult, indeed, is the chemical examination of organic substances, that we are continually finding chemists of the highest talents attributing to some of the fluids principles which are solely and altogether the result of their experiments in the laboratory. For example, *Tiedeman* and *Gmelin,* so celebrated for their analyses in organic chemistry, have admitted into the composition of the bile substances or principles which *Dumas* subsequently has most distinctly shewn to be generated during the analytic operations to which they have been subjected.

Again, it is so difficult to reproduce phenomena altogether as Nature has exhibited them, that often the substance which seems alike to our senses and our tests similar to another is, in reality, of a very different nature. Thus the (alleged) colouring matter of the blood, *hæmatosine,* separated by chemical means does indeed exhibit characters which have led the most distinguished chemists to believe that it is to its presence that the peculiar colour of this vital fluid is owing;—and yet it does not redden on exposure to the air, or even to pure oxygen. It cannot, therefore, be quite the same substance as that which exists combined with the other elements of the blood ...... . We cannot, indeed, wonder much at the imperfection of organic chemistry, seeing that it is only by analysis that we can discover the nature or composition of organised substances; almost all attempts at the synthetic test having failed."

So much for the uncertainties of the results of chemistry applied to the discovery of organic bodies and of organic operations.

M. *Andral* proceeds next to point out the extreme difficulty of drawing accurate conclusions in physiology from experiments on living animals.

"In physiology," says he, "the difficulties increase; another element, which we have not to guard against in animal chemistry, arises here; the chemist has to do only with dead matter, whereas in this new department of natural science the body is still under the influence of the vital power. All the phenomena of a living animal body—at least among the higher classes of the scale—are so linked together, that you cannot touch one without making the others more or less suffer and sympathise. Do you try an experiment to detect a phenomenon which is carried on *au sein* of a living body? All the great functions are at once deranged in their mode of manifestation; more or less blood is lost; it no longer follows in the vessels its regular and normal course, and it becomes either quickened or retarded in consequence of pain and fright; the secretions are immediately altered; at the part where the incision is made, there is an afflux of the fluids and of sensibility; and that great vital property, excitability, is at once brought into play; in short, you induce a state of active hyperæmia, or inflammation.

Wherever you touch a living animal being, it is the nervous system which responds by disordered movements, and by alterations in the sensibility, and which thus at once disarranges the phenomena which you wish to produce. Hence the phenomenon, which you represent as isolated, is not in fact so; and for this reason, that no point can be touched without causing a greater or less amount of disturbance of the entire organism.

........ It is not, however, to be denied that, in certain cases, experimentation has thrown considerable light upon some points of physiology, and also of pathology. Thus, for example, the experiments on the Fifth pair of cerebral nerves have afforded the point 'de depart' of our knowledge of the morbid lesions of these nerves; we could not have known any thing on this subject, unless we had the data which experiments have supplied us with. It is by them also that the special functions of the Portio Dura have been discovered, and that certain pathological facts connected with these functions have been understood and explained. In other cases, experiments on animals have been found to induce a train of morbid phenomena which are indisputably analogous with those which occur during certain diseases. Thus the injection of pus into the veins of animals has been observed to give rise to symptoms and appearances, which bear a striking resemblance with those exhibited in cases where a lesion of the blood has taken place spontaneously, as in scurvy, certain cases of typhus, the absorption of purulent matter, &c. Again, by experimenting we are enabled to study a certain number of the causes of diseases, as, for example, all the class of intoxications or poisonings—including under this term the introduction into the circulation not only of the common poisons from the mineral and vegetable kingdom, but also of miasms and of the various kinds of animal virus.

............ We find also, that by modifying the relative proportion of the constituents of the blood, various morbid states, which have their analogues in disease, may be induced. Thus, by withdrawing a certain quantity of the fibrine of the blood, we promote the effusion into various tissues; whereas by increasing its viscosity, a series of phenomena of an altogether different character is induced; as has been distinctly proved by the recent experiments of *Majendie.*

........ There is, therefore, a certain number of pathological lesions which may be explained by the results of direct experimentation.

We can excite inflammation and suppuration; but we cannot induce the formation of false membranes, of tubercles, of cancer, of hydatids, &c. Some specific cause or action is necessary to give rise to such morbid productions.

Assiduous attention, however, to various circumstances which are daily presented to our view, will probably enable us at length to advance in this obscure department of pathology. Already we know that the generation of hydatids in sheep is certainly promoted by feeding on wet pasturage."......

With the following brief remarks on certain necroscopic fallacies, we shall close our extracts from M. *Andral's* lectures for the present.

.......... "During hot weather, we not unfrequently observe on the lining membrane of the heart and great bloodvessels a well-marked redness, and the appearance of high vascular injection, which is the result not of an inflammatory action, as has been too often supposed, but altogether of the changes in the fluids and solids produced by incipient putrefaction. There are not a few pathological works which, I venture to say, are 'frappés de nullité,' from authors not being aware of this and such-like occurences.

For example; it is generally admitted that, under the influence of malignant fever, the tissue of the spleen is usually more or less extensively altered. Now, the late M. *Bailly*, of Blois, whose premature loss to medical science every one must regret, has, it is well known, published some valuable works on malignant intermittent fever; and in these he has particularly dwelt upon the remarkable changes in the spleen; such, for example, as the soft diffluent disorganization of its tissue. But in all his observations no mention is ever made either of the lapse of time after death when the dissection took place, or of the heat of the weather at the time. Now, this very state of the spleen is found in every body, if the examination does not take place, during Summer, within thirty hours after decease. We cannot, therefore, rigorously adopt the statements of M. *Bailly*, as we are ignorant of the circumstances in almost every case which he has reported."—*Gazette des Medecins.*

---

### NEW SORT OF MEDICAL PUFFING.

We are at present in quite a *puffing** epidemic. Here is an ingenious one.

Suppose that you visit Egypt, and there see a little negro rascal, whom his owner is willing to sell for a bottle of champaigne On your arrival in Paris, a short note makes its appearance in the corner of some journal, that a celebrated physician has recently brought over an interesting Ethiopian boy, whom he is educating at his own expense! A year afterwards, you convert him to Catholicism—a task which must indeed be very difficult;—a second puff for the christening!! Next year, he takes the sacrament for the first time: a third puff for this!!! Not long afterwards he marries; a fourth puff for the marriage!!!!

Now all this is very amusing, and withal very innocent; and the best part of the joke is that it may not be altogether unprofitable.—*Ibid.*

---

### SULPHATE OF QUININE IN ENLARGEMENT OF THE SPLEEN, AND IN DROPSIES AFTER AGUES.

Dr. *Levy*, physician of the Military Hospital of Val-de-Grace at Paris, has very satisfactorily shewn that the dropsical effusions, which not unfrequently supervene upon long neglected agues, are most successfully treated with quinine. He alludes to the researches of MM. *Bally, Nonat, Piorry*, and other contemporaneous physicians, which have clearly established the superior efficacy of this remedy—to be associated in most cases with the application of the cupping

---

* The French seem pleased with this word, for which, we suppose, they have no exact synonim, and which they have therefore adopted into their own 'belle langue.'

instruments over the left hypochondrium—in dispersing the enlargements of the spleen which so very generally, nay almost always, accompany old intermittent fevers. Indeed, he regards this, the administration of quinine in such cases, as one of the most valuable therapeutic discoveries of recent times. Now, as the dropsical effusions are almost invariably connected with an *infarcted*—to use an old word—state of the spleen, it is a rational deduction to anticipate that the remedy, which is so decidedly efficacious against the *cause*, may not be without its influence upon the *effect*. Certain it is that the use of the ordinarily-resorted-to means, such as diuretics and purgatives, seldom succeeds in dissipating the dropsies which we are at present considering, whereas they may be often dispersed by quinine in full doses.—*Gazette Medicale.*

---

### On the Remedial Power of the Lactate of Iron.

This new preparation of steel has already been extensively tried by some of the leading hospital physicians in Paris, and has met with their unqualified approbation.

The following extracts from a memoir by MM. *Gelis* and *Conté* will be sufficient to introduce it to the notice of the English reader.

" Several reasons have induced us to select the combination of the protoxide with the lactic acid: this acid is widely diffused through the economy; there is, perhaps, not one part of the body which does not contain a notable quantity of it. *Berzelius* has detected it in muscle, in milk, and in all the secretions; the perspirable matter owes its acidity to its presence, and a considerable quantity is found in the urine. The solvent power of the gastric juice is perhaps mainly attributable to the presence of the lactic acid: the traces of the hydrochloric are, it is now generally admitted, very feeble.

It must, therefore, be the lactate that is formed in the stomach, when any steel medicine is swallowed.

It is easily prepared by treating iron filings with diluted lactic acid. The water is decomposed, hydrogen is evolved, and the oxygen combines with the iron. When the evolution of the gas ceases, the solution is filtered and then evaporated until a pellicle forms on the surface: the salt chrystallises on cooling.

The lactate is not very soluble in water, and a high heat decomposes it. It is not readily affected by exposure to the air.

It may be administered in the form of pastilles, drops, or lozenges: the sugar which enters into the composition of these prevents the further oxydation of the salt. The dose is from four to fifteen grains.

The authors adduce several cases of chlorosis and other states of the system which are usually relieved by steel medicines, drawn from the practice of MM. *Bouillaud, Rayer, Beau,* &c. in which the lactate was administered with excellent effects: in some it succeeded after the usual ferruginous preparations had been fairly used without benefit.

M. *Bouillaud* has reported most favourably of this new medicine to the Academy: he has used it in 21 cases, and in all it produced excellent effects. It seems to have a marked influence in increasing the appetite. The dose was from six to 15 pastilles (five centigrammes in each: perhaps each patient took eight or ten grammes in all.

Professor *Fouquier* confirmed the favourable report communicated by M. *Bouillaud*. He stated, as his opinion, that the lactate would become one of the most valuable and standard ferruginous preparations used in medicine.

### ON THE FREQUENCY OF THE PULSE IN NEW-BORN INFANTS.

"We are astonished, upon examining the statements of *Billard* on this subject, to remark the very striking difference which he found in the frequency of the pulse in different infants of the same age, and in equally good health. Can we, for example, well admit that there should ever be a difference of 100 beats in the course of a minute? And yet this conclusion is deducible from the researches of this author.

In a recent work of M. *Jacquemier,* it is stated that the minimum of frequency of the pulse in new-born infants is about 97, and the maximum about 156. *Haller* fixed it at 140, and *Sæmerring* at 130. Every physician is well aware how easily the pulse of infants is rapidly quickened by their restlessness, during crying, and so forth; and that the only accurate way to determine it is to feel it during sleep. During the act of sucking, the breathing is hurried, and the circulation is therefore necessarily quickened at the same time. M. *Valleix,* who seems to have used the greatest precautions to avoid all sources of mistake, states as the results of his examinations, that in infants from 2 to 20 days old the minimum of frequency is about 76, and the maximum about 104. We may therefore consider the medium 87 as the expression of the average frequency of the pulse at this period of life.

It has been generally alleged that the frequency of the pulse diminishes as the age of the infant advances. The very reverse seems however to be the case, according to the experience of this author; for he found that at seven months, the pulse is much more frequent than during the first week after birth, and that from that period—seven months—it progressively diminishes in rapidity to about the sixth year or so.—*Clinique des Maladies des Nouveau Nes, par F. Valleiz,* 1839.

---

### PROFESSOR AMMON ON THE TREATMENT OF IRITIS.

In addition to vigorous antiphlogistic remedies, the "Archiater" of the King of Saxony, in his recently published Commentatio de Iritide, recommends the local use of belladonna in fomentations or in the way of friction on the temples and eyelids, and the internal administration of one or more of the following remedies—conium, arnica, colchicum, sarsaparilla, tartrate of antimony, red sulphuret of antimony, calomel, corrosive sublimate, the red precipitate of mercury, the muriate of barytes, turpentine, cod-liver oil, hydriodate of potash, &c., according to the severity of the disease and the constitution of the patient. If the constitution be scrofulous, he thinks highly of the muriate of barytes; if tainted with syphilis, provided it has not already been injured by mercurial indication, he prefers the corrosive sublimate; and if decidedly rheumatic he strongly recommends the tartrate of antimony in the dose of two or three grains daily; he mentions also colchicum as occasionally useful under such circumstances.

The author describes with great minuteness a variety of iritis which he denominates "seroso-cachectic," from its occurring in persons whose constitutions are unhealthy and cachectic.

"It is characterised by an obscurity of the cornea, loss of colour in the iris, and the accumulation of a white substance in the anterior chamber; the posterior surface of the cornea resembles an ulcer, its obscured centre projects out, and the conjunctiva becomes injected and swollen around the cornea.

The disease lasts for months or even for years. If the cornea regains its transparency, we find the iris colourless, and the pupil motionless: the vision is impaired or entirely lost from the beginning of the disease, and there is great sensitiveness to the light, and frequent supra-orbital pains. The treatment is generally difficult, and to be successful must always be to a great extent constitutional; saline baths, sarsaparilla, arnica, senega, and especially the hydriodate of potash or of soda are the best remedies."

What he says as to the management of syphilitic iritis may be comprised in a few words. If the patient has taken little or no mercury, the disease will be almost infallibly arrested by the judicious employment of calomel or of the corrosive sublimate; but if he has already taken a good deal, the preparations of this active mineral will be found to exasperate the mischief; in the latter case the *hepar sulphuris,* the hydriodate and carbonate of soda or of potash, sarsaparilla, and the extract of chelidonium should be resorted to. When syphilitic iritis occurs in a person of a scrofulous constitution, mercurials should be employed with great caution; hemlock, senega, sarsaparilla, and cherry-laurel water are often very beneficial.

According to the experience of Professor *Ammon,* one of the most intractable forms of iritis is that which sometimes follows the sudden retrocession of tinea in a scrofulous patient: the iris readily becomes atrophied, blood is effused into the anterior chamber, the inflammation spreads to the choroid coat, the ciliary processes and the capsule of the lens, and blindness is ultimately induced by a glancoma or atrophy of the eye being induced. In such a case antimonials and mercurials are hurtful; the most useful remedies are barytes, conium, iodine, &c. and the use of local blood-lettings and of revulsive applications.

When iritis occurs in arthritic patients and in women whose health has become cachectic at the change of life, Professor *Ammon* recommends the administration of the decoction of Zittman, the carbonate of soda along with the extract of taraxicum; and if the constitution be decidedly rheumatic, the use of turpentine, of colchicum, and occasionally also of quinine.—*Schmidt's Jahrbucher.*

---

### On Incontinence and Retention of Urine in Old Men.

The object of a memoir, which was recently read before the Academy of Sciences on this very troublesome affection of old age by Doctor Mercier, is to shew that it is in almost every case attributable to an enlargement of some portion of the prostate gland, and that it is very rarely or never dependent upon a partial paralysis of the bladder, as is generally believed, unless indeed there be paraplegia at the same time.

The author explains in the following extract the mode in which this enlargement acts in inducing incontinence.

........ "The neck of the bladder is not closed so much by a contraction of every point of its circumference, as by the apposition and coaptation of the two lateral halves of the prostate; thus it represents a fissure directed from before backwards.

By the antero-posterior hypertrophy of these lateral lobes, this fissure increases in length; but as long as its edges remain in complete approximation, the urine will be retained as in health; so that the gland may be very considerably enlarged without any notable disturbance in the excretion of urine.

But let us suppose that the edges are slightly separated from each other at one point; the result of this will be that the urine will escape; and if the separation be permanent and considerable, then there will be a complete incontinence. This is precisely what takes place in a multitude of cases."

He next alludes to the effect of enlargement of the third lobe of the prostate.

........ "Suppose that any substance is interposed between the edges of the neck of the bladder at its posterior extremity, so as to keep them apart, then instead of a fissure we shall have a triangular-shaped opening, the apex of which will be directed forwards, and through which the urine will escape the more easily, as the separation of the lateral edges is the more considerable, and as their approximation is less perfect. Now this is the effect of hyperthrophy of the third or middle lobe of the prostate; and it is this very condition of the parts which is by far the most frequent cause of incontinence of urine in old men.

This portion of the gland, which in the healthy state is so small that it can be scarcely demonstrated, often acquires a great enlargement in old age; and as this enlargement takes place in the transverse direction, the lateral lobes become more and more separated from each other. Whatever be its form, if it do not cover the orifice of the urethra like a valve, it induces a disposition to incontinence, which will be the more or less decided, according to the degree of separation of the lateral lobes and the density of the hypertrophied tissue; for if the tissue be soft, it will more readily yield to the muscular powers which compress it."

The author proceeds to shew that retention as well as incontinence of urine may be caused by the hypertrophy either of the transverse or of the lateral portions of the prostate. The following explanation of the mechanism in which the hypertrophy of the middle lobe gives rise to retention is ingenious:

........ "We have already seen that by increasing from one side to the other, the middle portion of the prostate has the effect of separating the lateral lobes, and consequently gives to the vesical orifice a triangular form which permits the urine to escape involuntarily. At the same time however it increases in other directions, chiefly forwards, where it forms a sort of valve transversely stretched above the posterior part of the neck. The more that this valve increases, the more are the lateral lobes separated posteriorly, and the more does the base of the triangle approach its apex. At length a time comes when they come in contact, and when the vesical orifice, instead of being an antero-posterior fissure as it is in health, becomes a curved or an almost straight opening from side to side. Then the valve on the posterior half of the cervix is in apposition with the anterior half, the orifice of the urethra is closed, and a retention of urine succeeds to a state of mere incontinence. Now such cases are of very frequent occurrence; and too often they are imagined to depend upon a paralysis of the bladder, their true cause being not even suspected."

An unequal enlargement of the lateral lobes of the prostate is apt to induce a certain degree of twisting or curvature of the urethral orifice to one side, and thus to give rise to a greater or less amount of dysuria: the introduction of the catheter under such circumstances is usually obstructed, and requires some tact on the part of the surgeon.

Dr. *Merceir*, although convinced that essential or idiopatic paralysis of the bladder is exceedingly rare, even in old age, admits that chronic inflammation of its mucous coat is apt to be followed by an impairment of the contractility of its muscular tunic.

Now as this state is very often co-existent with enlargement of the prostate, the dysuria under such circumstances must be necessarily aggravated.

Dr. M. quotes the following passage from *Sir A. Cooper's* Lectures on the Principles and Practice of Surgery, 1835, p. 482, to shew how mistaken some of the most eminent surgeons are as to the true pathology of retention and incontinence of urine:...... "retention of urine, when it is incomplete, must be considered as salutary, since it prevents incontinence which would otherwise constantly take place in old men."

"Now I ask," says Dr. Mercier "would Sir *A. Cooper* have held this language, had he been aware that these two affections, so different, are owing to the same cause? *When an old man has difficulty in retaining his urine, and finds himself obliged to let it escape on the very first call, we have strong reason to fear that sooner or later he will have retention till at length he is quite unable to void it.*"

The following four propositions embody the substance of our author's ingenious observations on these very common and very distressing maladies of old age.

1. Except where there is a disease of the brain or of the spinal marrow, or a general prostration of the entire system, incontinence and retention of urine in old men depend almost exclusively on an hypertrophied state of the prostate: it is for this reason that the one affection so often succeeds the other.

2. The more that this hypertrophy affects the gland in an equal and regular manner in all its parts, the greater will be the disposition to incontinence.

3. On the contrary, the more partial and irregular the hypertrophy is, the more imminent will be retention.*

4. It is in cases which are intermediate between the two categories now mentioned that the state of overflowing (regorgement) of the urine is most apt to take place.—*Gazette Medicale.*

---

### Prolapsus Recti cured by a New (?) Operation.

A woman, 33 years of age, had the misfortune to become affected during her third pregnancy with a prolapsus of the rectum, which was however only temporary, and did not occasion a great deal of distress. During her next pregnancy the prolapsus became much more considerable and was permanent; moreover it was accompanied with a descent of the uterus, and with a great relaxation of the abdominal parietes.

M. *Roux* excised a ring or circular portion of the mucous membrane of the prolapsed gut: this operation afforded relief for some time; but the prolapsus again began to increase, the discharge of the alvine contents became involuntary, and such severe pains were felt in the loins and thighs, that the patient was obliged to keep her bed. In this state she was admitted into the Hôpital de la Pitié under the care of M. *Robert.* The sphincter was found to be so much relaxed that four fingers might readily be passed into the gut.

It was proposed to excise a portion of the sphincter proportioned to the amount of relaxation; but before proceeding to the operation the patient was put upon a very restricted diet, and opium was given to ensure a prolonged constipation of the bowels. This being effected, M. *Robert* made an incision on each side of the anus, the two converging to the point of the coccyx. The flap of integuments between the two incisions, comprising a portion of the sphincter muscle also, was detached, and the edges of the wound were then brought and kept in apposition by three points of the twisted suture.

On the sixth day after the operation the sutures being removed, the wound was found to be nearly united. On the fifteenth day no alvine evacuation had as yet taken place; but as the desire to relieve the bowels was very distressing, the fæces were removed with a scoop.

On the fortieth day the patient, who before the operation could not retain at all the contents of the bowels, kept an enema an entire day: there was no longer any prolapsus; the anal orifice had recovered its normal dimensions, and did not admit readily more than one finger, and the patient was able to relieve the bowels at will and without distress.—*Gazette Medicale.*

---

* This opinion is adopted by M. *Leroy d'Etiolles* in a memoir which he recently submitted to the Academy, and wherein he says:—" In all these patients the complete or incomplete retention proceeded from a partial tumefaction of the prostate. The uniform swelling of this gland, without retention of urine, existed in three patients."

### A NOVEL MONSTROSITY—PORTION OF A FŒTUS DEVELOPED AT THE EXPENSE OF THE TESTICLE.

The following case is certainly one of the most extraordinary in the records of surgery, and cannot, as far as we know, be exactly parallelled. What are we to think of a portion of a fœtus being fixed in the testicle of a man, where it seems to have been developed since the period of birth! The truth of such a statement might indeed have been fairly questioned, had the case not been examined by dozens of medical men, and if there was not the *material* proof in the preparation which has been laid before the Academy of Sciences.

A man, 27 years of age, was admitted into La Charité Hospital in consequence of a tumor situated on the right side of the scrotum. It was of about the size of the fist, firm and unyielding, and seemed to be quite distinct from the substance of the testicle: its enveloping integument too presented a very different appearance from that of the skin of the scrotum. When examined by the fingers, it felt irregularly knobby or lobulated; and was so insensible that it might be pricked and even cut without causing any pain to the patient. From an ulcer on its posterior surface a small tuft of hair had come out, and from another on its anterior surface some glairy grumous matter had several times been squeezed out. The consideration of these various circumstances suggested to M. *Velpeau* the idea of a *fœtal growth,* or a *product of conception,* although several surgeons had regarded the tumor as of a fibrous or cancerous nature.

The following is the account given of the tumor after extirpation :—

" The examination of this tumor has enabled us to discover within its substance the presence of almost all the anatomical elements of the body of a mammiferous animal. Thus, its external or enveloping stratum was evidently of a cutaneous nature; its principal substance was a *melange* of laminæ and fibres, which suggested the idea of cellular, adipose, and muscular tissues. In the interior, we found two small cysts whose contents resembled albumen or the vitreous humour of the eyeball; another cyst, which was as large as a partridge's egg, contained a matter of a greenish-yellow colour and of semi-fluid consistence like meconium; in a fourth sac there was a grumous mass of a dirty-yellow hue, concrete and surrounded with hairs: this matter, when examined with the microscope, exhibited all the characters of sebaceous matter and of epidermic scales or laminæ. From one of these cysts, that one which was filled with the greenish matter, came the tuft of hair; and the opening, from which this came out, had some analogy with an anus. Imbedded in all these elements were numerous portions of a skeleton which were perfectly organized, and which indubitably belonged to *veritable* bones and not to merely accidental productions. These bones, which were all invested with a periosteum, and which were not only moveable upon each other, but exhibited also genuine articulations, were divisible into three groupes: the first groupe consisted of three pieces which seemed to be the clavicle, the scapula, and a portion of the humerus; the second groupe, much larger than the former, seemed to belong either to the pelvis or to the base of the cranium, according as we might consider the central portion to be the body of the sphenoid bone or the sacrum; and the third groupe seemed to comprise portions of the vertebræ or fragments of undetermined bones."

But however uncertain may be our conjectures as to the classification of the different bones, we cannot for a moment doubt but that they belong to a product of fecundation, in other words to a fœtus already considerably advanced in development. The fact cannot be denied, however difficult it may be to afford any explanation of it.

In that variety of monstrosity, designated *monstrosity by inclusion,* which consists in the development of one fœtus within some part of the body of another, the inclosed fœtus is always found to be surrounded or enveloped with a cyst, so that it is, as it were, a foreign substance imbedded in the tissues of the fœtus which has continued to live. In the examples related by Saint *Donat, Prochaska, Dietrich,* &c., where there were the appearances of fœtal debris in the testicle, it may fairly be a question whether in such cases the tumor was not of an encysted nature, or produced by some organic parts in a state of decomposition; but certainly in the case related above, every thing on the contrary had continued to live. The abnormal tumor had its own colour, its own consistence, and its own sensibility, quite independent of the body which supported it: a distinctly marked line separated its teguments from the skin of the scrotum. "I have pinched it most severely, and pricked it with various instruments, and the youth has himself repeatedly forced the blade of a pen-knife into it without causing any pain! the wounds however always bled freely, inflamed, and cicatrised like those of any other organised part: there was no other sign in it of an abnormal condition."

One curious circumstance in the history of this case is, that the tumor must have grown considerably from the period of infancy, as the surgeon who saw it about four months after birth, considered it merely a pneumatocele, and paid little attention to it, and afterwards he called it a small phlegmon which would terminate in resolution.

There is reason, therefore, to believe that the tumor continued to increase gradually in size to the fifth or sixth year of age, and that after this period it remained stationary.

M. *Velpeau* closes his narrative of the case in the following words:—

"Is it possible that during intra-uterine life a part of a fœtus, the rest of which had disappeared, should become glued, as it were, to the scrotum, so as to remain as a graft or bud? or can we suppose that the fœtus had primarily been developed in the abdomen of the other, and then descended through the abdominal ring into the scrotum, and subsequently underwent in its new position a certain change and decay? or lastly, can we regard the tumor and its contents as a creation on the part of the testicle? But I must stop in my conjectures, as these are questions of profound physiology and of transcendental anatomy which it would be unwise to approach, until the specimen is carefully examined by the master of the academy.—*Gazette Medicale.*

It may be interesting to give a brief abstract of the celebrated report of Dupuytren in 1812, on a remarkable case of a fœtus being found in the mesentery of a youth 14 years of age. The boy had always been in delicate and feeble health; from his infancy he had suffered from a pain in the left side of the chest and abdomen; and there was a swelling at this part, which was suspected to be of the nature of an encysted tumor. When residing at a boarding school near Rouen, he was seized with fever, attended with a violent pain and tumefaction, extending from the left hypochondrium down to the pelvis. On the seventh day after the attack, his physician felt in the left flank a hard tumor painful to the touch, and seemingly as large as a full sized melon. Gradually his sufferings abated, as a diarrhœa of fœtid purulent matter came on. His strength by degrees sank, and he died in a state of complete marasmus. Six weeks before his death he had voided by stool a large tuft of hair.

*Dissection.* In the left hypochondrium was a large pouch or sac, adhering closely to the walls of the colon. Within were contained two principal masses; one of which was entirely composed of a large handful of densely interlaced hairs, and the other consisted of a fleshy osseous mass invested with skin: at one of its extremities there was a deformed head with an imperfect nose, eye, and ear; and at the other an appendix in the form of a limb terminated by several *languettes* which were provided with nails; from the centre of the mass, which seemed to occupy the place of the chest and abdomen, there proceeded a thick short ligament to be inserted into the walls of the cyst. The organized mass was three inches and a half long, and nearly three inches across. It was sent to Paris, and carefully examined by a committee, consisting of MM.

*Dupuytren, Cuvier, Richard, Baudelocque* and others. The skeleton was found to be composed of a spinal column, a pelvis, a cranium, rudiments of limbs, and six teeth placed around a sort of mouth ; there were no traces of an œsophagus, stomach, lungs or heart; the brain was converted into a hard bony substance.

*Dupuytren* has recorded that, in addition to this very curious case, he has twice met with the debris of a fœtus in the uterus of young girls, twelve years of age, and in whom the possibility of pregnancy was entirely out of the question.

The Journal of Geneva for 1775, mentions a curious case where a cyst exhibiting all the appearances of a uterus, and which inclosed a well formed fœtus, was found in the abdomen of a man who died of dropsy.

To explain the presence of fœtal debris in certain tumors, we must admit one of two hypotheses:—either that of two germs primarily distinct from each other, one has penetrated and been admitted into the other from some cause which we cannot explain, or that they were joined together from the very first in the same ovulum or germ.

The first hypothesis has been adopted, we believe, by St. Hilaire; the second by *Himly* in his scientific work (Geschichte des Fœtus in Fœtu, 1831), and *Santo Fattori;* and certainly receives support from the known fact that we occasionally meet with two germs in the same seed-grain, and also in the same bird's egg.

Whichever opinion we adopt, we must admit that one of the fœtuses becomes somehow inclosed within, and grows as it were, at the expense of the other; hence the latter has been appropriately termed by *St. Hilaire* "parasite," and the former "autosite."

The *parasite* is usually enveloped in a membrane which adheres strongly to some part of the *autosite,* and in which the anastomosing vessels which connect the two beings ramify and inosculate. In no instance has a heart or any organ, which could carry on the circulation of the parasite independently of the autosite, been ever found.

Hair, teeth, and fatty matter in great quantities, form usually the greatest bulk of the parasitical growth; and these structures require, it is well known, a small amount of vitality for their production.

M. *St. Hilaire* has grouped the various *monstrosties by inclusion,* (as they have been termed) into two classes—the external or those situated under the integuments, and the internal or those situated in one of the visceral cavities.

Of 19 cases of external parasitical growths, recorded by different authors, one was situated in the neck, one in the epigastrium, one over the pubes, eleven over the sacrum, and five over the scrotum.

Of those situated in the sacral or sacro-perineal region, most were moveable, fluctuating, and well-supplied with blood-vessels; in some of the cases they were felt to contain a hard substance or substances within : such tumors have been known to hang down as low as the knees. In most of these cases, the *autosite* fœtus or child has had some other congenital malformation, such as imperfect development of the limbs, hydro-rachis, &c. and has seldom survived long after birth.

Of 22 cases of internal parasitical growths, eighteen have been found in the abdomen, one in the anterior mediastinum, one in the ovary, and two in the uterus, before conception was possible. In the abdominal cases, the tumor has always been found to be external to the peritoneum ; and most frequently it has been situated in the upper part of the abdomen. If such be always the case, the diagnosis of such tumors from extra-uterine conceptions may be assisted by attending to these two circumstances.—*Bulletin de Therapeutique and Bulletin Med. Belge.*

---

### M. Guerin on Subcutaneous Orthopœdy.

We are informed by this indefatigable reformer of deformities that within the last four years he has performed upwards of a thousand operations for the section of muscles or tendons in different parts of the body.

The following catalogue of muscles divided he gives in the number of the French Medical Gazette for last May.

1. In the neck—the sterno-mastoideus, cleido mastoideus, trapezius, angularis scapulæ, splenius, complexus colli, and cervicalis descendens.

2. In the back—the trapezius (along its entire attachment to the scapula), rhomboidens (along its entire attachment to the scapula), dorsalis magnus, sacro-lumbaris, longissimus dorsi, and transversalis dorsi.

3. In the upper extremities—the deltoideus, biceps, supinator longus, radialis anticus, ulnaris anticus, flexor sublimis, and extensor digitorum communis.

4. In the lower extremities—The psoas and iliacus, (*psoas-iliaque)*, adductor-longus, sartorius, rectus anticus, tensor vaginæ, glutei, biceps, semi-tendinosus, semi-membranosus, rectus internus, tendo-achillis, tibialis anticus, tibialis posticus, flexor communis longus et brevis, flexor pollicis longus et brevis, extensor longus communis, extensor pollicis, peronei anticus et laterales, plantaris, adductor and abductor digiti minimi.

5. Aponeuroses—fascia lata, and plantaris.

6. Ligaments—sterno-clavicular, scapulo-humeral, coxo-femoral, lateral of the knee, tibio-astragular, lateral and posterior astragulo scaphoid capsule, and scaphoid-cuneiform capsule.

---

### On the Subcutaneous Incision of Joints.

In a former memoir on subcutaneous wounds—vide the last number of the Medico-Chirurgical Review—M. *Guerin* has shewn that, as long as wounds are kept quite excluded from the contact of the air, there is seldom any tendency to inflammation set up, while the healing of the divided parts is usually effected very rapidly.

Hence the importance of performing certain operations without making more than a mere puncture of the integuments. In his second memoir the author proceeds to shew by experiments on men and on animals, that the subcutaneous incision of joints is as exempt from danger as that of tendons, muscles, nerves, and small blood-vessels, and moreover, that it may afford in certain cases a valuable means of cure in the hands of the scientific snrgeon.

In two dogs he opened successively, by the subcutaneous method, the humero-cubital, the radio-carpal, the femoro-tibial, and the tibio-tarsal joints; and he found that, whenever the wounds were kept quite excluded from the admission of the air, they rapidly healed without any inflammation.

When the joints thus opened were allowed to be moved about, a synovial swelling usually appeared around the wound, but when they were kept quite quiet and extended, even this trifling accident did not supervene. If however the air was permitted entrance, inflammation and subsequent suppuration were invariably induced.

M. *Guerin*, reasoning upon the results of these experiments as well as upon what is well known to happen after some severe dislocations, very rightly inferred that there cannot be any essential danger in making an incision into a joint, provided the air be excluded from the synovial cavity. He has now in several cases divided the ligaments and portion of the capsules of the knee and ancle-joints; and in none of these operations has any unpleasant symptom manifested itself.

The wound in the integuments should always be as small as possible, and far distant from that into the joint: moreover it should be made when the limb is extended, and never when it is bent; and lastly, the joint should be kept perfectly motionless for some days afterwards.

"These two last-named injunctions, are," M. *Guerin* observes, "the more necessary to be attended to, as I have discovered recently that in all the movements of joints their cavities become more or less enlarged, and therefore that as a vacuum is thus formed, there is a strong tendency to the suction of air, if any communication exists with the outer surface."

In the third part of his memoir, the author points out the useful applications which may be made of his researches to the improvement of surgery. Serous, sanguineous, or purulent collections within the joints may be evacuated without danger. Among the most extensive and important applications, he mentions the subcutaneous division of articular ligaments and capsules, with the view of maintaining in a fixed condition certain congenital and also some old dislocations after they have been once reduced; also the exciting of adhesions, and favouring the formation of new articular cavities.

Already he has effected in this way the cure of a congenital luxation of the clavicle which had resisted every means that had been tried, by making numerous sections of the ligaments all around the displaced joint.

---

### ON CONGENITAL SUBLUXATIONS OF THE FEMUR.

The following are the conclusions which M. *Guerin* has drawn from all his researches on this question.

1. Congenital luxation of the femur is, like club-foot, wry-neck, and deviations of the spine, the result of primary muscular retraction ; and the various forms of this luxation considered as to their seat, their direction, and their degree, are produced by the muscular retraction being differently distributed, and by its elements being differently combined, in the muscles of the pelvis and of the thigh.

2. There is an order of congenital deformities of the hip, which has not been noticed by any writer, and which I have called *pseudo-luxations,* because they present the deceptive appearance of luxation without the escape of the head of the bone from its socket;—this also is the result of muscular retraction.

3. The essential treatment of these deformities, independently of the means already known, consists in the section of the retracted muscles. I have already performed this operation three times with success.

These remarks are equally applicable to the congenital luxations of other joints. The essential cause of all, wherever they are seated, is what we have stated above—viz. primitive retraction of the muscles inducing shortening, partial paralysis, and arrested development of their tissue. A few weeks ago I divided the biceps, semitendinosus, semimembranosus, and rectus internus muscles in a case of incomplete luxation of both knees, which occurred in a girl fourteen years of age : on each side there was a sub-luxation of the tibia backwards upon the condyles of the femur, a considerable rotation outwards of the leg, and also an inclination outwards of the leg upon the thigh of nearly 60 degrees. On the day after the operation the outward rotation, the lateral inclination, and this displacement backwards of the tibia were no longer perceptable, and there was merely a certain degree of flexion of the leg upon the thigh : the cure was complete, with the exception of this partial flexion remaining permanent.

To give confidence, adds M. *Guerin,* to those who have not had an opportunity of witnessing the *innocuous nature of subcutaneous operations,* I may state that the other day, in the case of a young girl, I divided thirteen different muscles or tendons for the relief of various deformities, and that from the following day she experienced neither pain nor *malaise*, nor any symptom of inflammation in the seat of the divided muscles.—*Histoire des Difformités du Systeme Osseux.*

---

### Operative Midwifery in Holland and Germany.

### *Case of Symphysotomy.*

A woman, 26 years of age, and who had been twice before delivered of dead children by means of the forceps, became a third time pregnant. Dr. *Gelauff*, who had attended her in her former pregnancies, *unwilling to expose himself to the recurrence of so much difficulty as he had met with before, and wishing moreover to deliver a living child*, resolved to perform the operation of dividing the symphysis pubis for the purpose of increasing the antero-posterior diameter of the inlet of the pelvis—which he ascertained to be less than three inches. The operation was performed between five and six o'clock P. M. we are told, according to the rules of art; and the bones were separated a little less than half an inch. The pains did not come on briskly for several hours afterwards; but about ten o'clock they increased so much that, very soon afterwards, the child was expelled alive. The placenta also came away as after a natural labour. The recovery was altogether so satisfactory that the woman was able to walk about at the end of the fourth week.

### *Cases of Cæsarian Operation.*

A woman, 32 years of age and seriously deformed from rickets, unfortunately became pregnant. At length the period of labour came on, and Dr. *Straek* of Cassel ascertained that the child was alive, but that the pelvis was so much contracted that no living child could pass through it. The operation of extracting the child by the Cæsarian section was therefore determined on. Having divided the abdominal parietes and peritoneum in the usual manner, an opening was made into the uterus and sufficiently enlarged to enable the operator to introduce his hand, turn and bring out the fœtus—the placenta also was easily removed.

The uterus immediately contracted upon itself, and expelled a quantity of coagula which were carefully wiped away. The contraction being deemed sufficient—half an hour elapsed for this purpose—the external wound was brought together by several stitches, and long strips of adhesive plaster.

The child, when put into a bath, cried lustily. The mother, in spite of two or three attacks of pulmonic irritation which required venæsection, ultimately recovered most satisfactorily. For the first few days she was able to suckle her child, but, the secretion failing, a wet-nurse was engaged.

Dr. *Hoebeke* of Brussels, whose extraordinary success in Cæsarian operations we gave a short account of in the Number of the *Medico-Chirurgical Review* for October of last year,* has appended to the above account the report of the following case which occurred in his practice so far back as the year 1829.

---

* We presume that it is the same gentleman, although in our preceding notice he is designated M. *Hoebecke* of Sottegem in Flanders. He has performed the operation thirteen times; ten mothers and nine children have been saved. The fatality of the operation in the practice of other medical men he attributes not

*Case* 2.—A poor woman, who was miserably rachitic, had been in labour for three days when Dr. H. was called to her assistance. He found that the antero-posterior diameter of the pelvis measured less than two inches. Various fruitless attempts having been made by the accoucheur in attendance to apply the forceps, and extraction *per vias naturales* being in truth impracticable, the Cæsarian operation was at once resolved on. The child was found to be dead, when taken out. The woman was completely cured, we are told, by the end of the third week. (!)

*Remarks.*—We have repeatedly condemned the unjustifiable disregard of maternal life in various countries on the Continent, as exhibited in the readiness with which the medical men perform the frightful operation of the Cæsarian section. Not only in Germany and Holland, but even in France, where the surgeons and physicians, to their credit be it said, are usually among the foremost to adopt any acknowledged improvement in all the departments of the healing art, is this operation much more frequently resorted to than with us, but it is often undertaken under circumstances which every unprejudiced person, be he medical or not, will surely not hesitate to condemn. For example, the pelvis of a woman may not be so well formed as to give passage to a full grown nine months' child, but yet be sufficiently ample to permit a seven months' child to pass; and as it is well known that children at this premature period are usually *viable* or capable of sustaining life, then why allow any poor deformed creature to go her full period of gestation, with the inevitable prospect of a dreadful operation before her eyes? We have more than once within the last few years alluded to cases where women have actually been admitted into hospitals in the sixth and seventh months of pregnancy at which period the deformed state of their pelvis has been ascertained, and yet no step has even been thought of to avert the horrid catastrophe impending over them. Nay cases have been reported where women, who have actually undergone the operation once, have been received again, in a subsequent pregnancy, to be subjected to it a second time!

Let us hope, however, that a more humane, aye too and a more scientific feeling will soon be manifested among our Continental brethren, and that they will be induced to imitate the example set by all the most eminent accoucheurs in Britain—we mean that of bringing on artificial labour at the seventh month or earlier, in cases of ascertained deformity of the pelvis. We perceive, by some of the late reports of the Academy of Medicine, that one of the leading obstetrical authorities in Paris, M. *Dubois*, has been acting on this principle in the case of a poor dwarf who had unfortunately become pregnant. Labour was induced at the beginning of the eighth month by means of a piece of sponge introduced into the os uteri, and of the administration of the ergot of rye,* both by the mouth and in enemata; the child was born alive, and the mother recovered most favourably.

---

so much to the dangers necessarily consequent upon it, as to it having been undertaken at inopportune times. The particulars of his extraordinary success will be found in a volume which he has recently published and entitled *Observations Pratiques de Chirurgie et d'Obstetricie.*

* We are surprised to find that so intelligent an accoucheur as M. *Capuron* should question the propriety of the practice of his colleague in this case. The only reason that he gives is, that he has known a case of a woman, whose height was short of three feet, giving birth to a living child at the full time. Surely it does not follow that, because she was so diminutive, her pelvis was not developed. As a matter of course, the size of the pelvis is to be ascertained before we resort to the induction of labour.

In a recent number of *Schmidt's Jahrbucher,* we find a report of a successful case of Cæsarian operation performed by Dr. *Michaelis* of Kiel*; and short notices of several other cases which have occurred in different countries within the last year or two. They are as follow :—

1. Cæsarian operation fatal, by Mr. Ward, (extracted from an English journal). The inlet of the pelvis was not more than one inch in its antero-posterior diameter, in consequence of the extreme projection of the sacro-vertebral angle; the fœtus was dead.

2. Cæsarian operation; extraction of a living child; death of the mother on the third day. Another fatal case is also recorded by the same gentleman, Dr. *Hamm;* and both are detailed in a late number of the *Neue Zeitschrift.*

3. Cæsarian operation successfully performed for the second time on the same woman, by Dr. *Foy.—American Med. Journal,* 1838.

4. Successful case of the Cæsarian operation, by Dr. *Bauer.—v. Journal de Siebold,* t. xvi.

5. Successful case by Dr. *Hertzbruch;* death of the child.—*v. Neue Zeitschrift.*

6. Successful case by Dr. *Wiefel.—v. Casper's Wochenschrift.*

7. Second history of a successful Cæsarian operation by Dr. *Schenk.—v. Siebold's Journal.*

8. Successful case by Dr. *Petrenz.—Ibid.*

To these cases we may add another in which M. *Dubois* performed the operation last March, at the Clinique des Accouchements in Paris; the child lived, but the mother died on the sixteenth day after the operation of tetanus, after the fairest promises of recovery.

In the report of this case it is stated that M. *Dubois* has now performed the operation *five* times: every one of the patients has died.

Contrasted with this, the success of the German and Dutch practitioners is indeed surprising. We read of the operation having been performed two, three, and even four times on the same woman! It seems almost incredible that any woman would expose herself to such inevitable suffering after having once undergone this most frightful operation; and yet from a report of M. *Michaelis* it appears that of 40 women who had recovered from it, 15 of them again became pregnant!

---

### On the Sweating Regimen of Dr. Priessnitz at Graefenberg.

For some years past a Dr. *Priessnitz* has acquired a great reputation over Germany for the success which has attended his practice for the cure of chronic diseases, and which has made his residence at Graefenberg visited by invalids from all parts of the Continent. Dr. *Bigel,* Knight of the Legion of Honour, and one of the Medical Professors in the Imperial College at Petersburgh, has recently published an account of this regimen, under the title, "Manuel d' Hydrosudotherapie, ou Traitement des Maladies par l'eau froide, la sueur, l'exercice, et le regime." He seems to be an ardent admirer of the sweating regimen as pursued at Graefenberg, and, as he has derived personal benefit himself from its adoption, we may attach the more credit to his statements. Dr. *Priessnitz* is evidently a disciple of the old humoral school of pathology—and we believe that in this respect he is far from being alone either among his own countrymen, or his professional brethren in England and France—for he considers almost all diseases to arise from the existence of some morbid or peccant humors generated

---

in, or introduced from without into, the blood; and his practice, founded upon this idea, essentially consists in using means to effect the expulsion of these humors from the system, and to replace them with healthy juices. These means are *water, air, exercise,* and *diet.*

The causes which, in his opinion, most frequently engender the unhealthy humors of the body, are the use of improper food, or the excess of what is wholesome, the suppression of perspiration, the want of due exercise, the influence of various mental emotions, especially such as are of a depressing nature, the inhalation of a noxious atmosphere, the neglect of cleanliness, the use of stimulating liquors, &c.

His remarks especially on the neglect, as well by physicians as by unprofessional people, of the use of baths and other means to regulate the state of the cutaneous functions, are exceedingly pertinent: "We every day," says he, "see whole families purged and vomited by the order of their physicians, but rarely or never do we hear of their being recommended to wash their skins."

No one can find fault with Dr. *Priessnitz*'s practice in this respect; in it there is certainly no want of washing both inside and outside man's 'muddy vesture of decay.'

"Ablutions," says he, "with cold water, are doubtless highly useful in promoting the cutaneous exhalation, a function so necessary for the preservation of health; and the drinking of cold water contributes not less powerfully to the same end, by preventing the *stasis* of the juices and impressing upon them a regular circulation. Again, it is no less important to expose as frequently and as freely as possible the body to the fresh air, since it is from this element that we derive the very principle of life, the oxygen. Just in proportion as there is less air, so there is less oxygen, and consequently the less of the principle of life is present." He insists very particularly upon the utility of drinking very largely of pure cold water. Copious libations of this element constitute a most important part of his regimen. In reference to other beverages, he says:—

"It is a great mistake to suppose that the use of pure cold water can be compensated by the drinking of tea, coffee, or thin beer, as is too commonly imagined; for it is not only as a diluent, and dissolvent of acrid matters in the bowels and kidneys, and as an attenuant of unwholesome juices in the blood, but also as a powerful means of communicating oxygen to the system, that pure water, taken in large quantities, acts so beneficially. In short, let any one try the effects upon him or herself of drinking freely and largely of the fair element, and he will not fail speedily to acknowledge that his health has been much improved."

M. *Priessnitz* is by no means a *starving doctor;* a fundamental principle of his treatment being to maintain the system in a state of vigorous strength. But then he is somewhat of an exclusive. He interdicts all spiced and highly-seasoned dishes, all hot peppers and other foreign spices, which may suit the natives of the countries where these grow, but which are not required in our climates where the air, by being more highly charged with oxygen, is more stimulating. He permits the free use of our own vegetable productions, such as our various culinary herbs and salads, including radishes, mustard and such like stimulants, provided the patient is free from gout and cutaneous disease.

Cold meats he prefers to hot, especially for persons with weak stomachs. For breakfast and supper by far the best articles of food are bread, milk and butter.

He does not restrict his patients as to the quantity of the food they take; Nature he thinks, will seldom err, in this respect, provided the appetite is not provoked by condiments or stimulating liquors, and the person takes sufficient exercise.

The grand principle of his treatment consists in supplying the system with the means of deriving healthy juices from the food that is taken, while, on the one hand, all the poisonous and peccant humors are evacuated by regular copious sweating effected in the manner which we are about to describe, and, on the other, the system is fortified by the use of cold bathing, used while the sweating continues.

Our readers will observe that his practice in this respect is entirely analogous to that of the Russians, who, it is well known, are in the habit, upon leaving their vapour baths, of immediately, and while the body is drenched with perspiration, rolling themselves in the snow.* Dr. *Priessnitz* has probably derived the hint from this custom of his neighbours.

The following is his plan of a *sweating bath.*

The person, stripped naked, is inclosed in a long blanket, which is then to be wrapped close round his head, body and limbs, (the arms are to be kept applied to the sides, or folded across the chest,) leaving no part exposed but his face. The ends of the blanket are to be tucked up, as nurses do with the clothes of an infant, and the whole is then to be secured with bands put round various parts and tied.

The patient thus hermetically cased in his blanket, is to remain quite quiet—he may fall asleep if he chooses—until the skin begins to sweat freely. This seldom happens before the expiry of one hour, and frequently for not a much onger time.

By endeavouring to move his limbs up and down, and by rubbing his body with his hands as well as he can in his mummy-like wrapping, the tendency to sweating will be increased.

As soon as this commences, the window of his chamber is opened, and he is to drink, every quarter or every half hour, a glassful of cold water. This increases the perspiration so much that it often not only oozes through the blanket but drips down on the floor. Sometimes not less than seven pounds may be collected in a vessel placed below the bed. The sweating is to be kept up for at least an hour; in certain cases for two, three, or even four hours. Some patients are subjected to this process twice in the twenty-four hours, at four o'clock in the morning, and again an hour or so after dinner.

When the sweating has been continued sufficiently long, the patient is then unswathed, and, covering himself with a cloak, he hurries to the bath, where, after first wetting his head and chest with the water, he plunges into the cold water, his body still streaming with perspiration. While in the bath, he should move about his limbs and exercise every part as briskly as possible, in order to promote a reaction in the cutaneous vessels.

Now this quick transition, from a state of profuse perspiration to the use of a cold bath ; is not attended with any danger, such as is often the case, if the body has been perspiring freely from violent exercise, or from the use of stimulating sudorifics. The important feature of M. *Priessnitz's* plan is, that the organs of circulation and respiration are not at all quickened or excited; they remain quite tranquil. Hence there is no sudden check given to these vital functions, and the body, instead of being chilled by the sudden transition, is invigorated and glows with warmth over its entire surface.

The greater that the cutaneous reaction from the use of the bath is in any case, the more favorable is the prognosis, and the more speedy will the cure of the existing disease be.

When the patient is unswathed to go to the bath, all that is necessary is only to drink a glass of water as often as he may feel himself much heated. For the same reason it is not requisite to wet the head and chest before plunging into

---

* The same impunity is observed in the eating of ices in tropical countries, when the body is perspiring freely, provided always the breathing is not quickened at the time, and gentle exercise is taken immediately afterwards.—*Rev.*

the bath, except when this (the bath) is situated at some little distance, and when the outward air may possibly have somewhat closed the open pores of the skin. Let no one be sceptical of the truth of these statements.

Any one, who has witnessed the patients at Graefenberg, will be at once satisfied that there is not the least danger in plunging into a cold bath while the whole surface is sweating most profusely, *provided the breathing is not hurried at the time.*

If attention be paid to this one caution, there is little or no risk of any injurious consequences from the immediate transition from a state of profuse perspiration to immersion in cold water. But if the surface is permitted to be at all chilled, and the perspiration is in any degree checked by unnecessary delay, before the immersion takes place, a portion of the internal heat of the system, which is requsite for its due reaction, is lost, and then unpleasant consequences are often observed to ensue.

We again repeat that if these two precautions—viz. the preventing a quickened state of the breathing, and a chilled state of the skin—were more uniformly attended to, we should hear of fewer accidents from bathing when the body was heated or perspiring freely.

On leaving the bath, the body is to be well rubbed; and the person then dresses himself and takes a walk for half an hour or upwards, to keep up a uniform glow over the whole system. During this promenade, it is useful to drink a tumbler-full or two of cold water.

In many cases the cold douche baths are used at Graefenberg with astonishing success, especially against chronic gouty, and rheumatic ailments. The patients always walk to them, for they are all situated at a considerable distance from the dwellings, exercise after their use being strongly recommended: during this exercise cold water is to be freely drunk.

There are various other ways in which cold ablutions are recommended by Dr. *Priessnitz,* such as the hip and foot bath, washing the head with cold water, and injections of it into the bowels. In short cold water is the sole and only remedy that is used in almost every case. No other application is ever applied to any abscesses or outward sores, than linen dipped in cold water and then covered with oil-silk or other means to prevent the evaporation.

The sweating regimen at Graefenberg induces, in very many cases, an eruption of boils on various parts of the body; these, when troublesome, are always treated with fomentations of cold water. Ointments are never used.

From the preceding remarks, our readers will see that the sweating regimen of Dr. *Priessnitz* is any thing but a feeble and ineffective practice. If there are peccant humors in the body when sick—and who will deny their presence?—and if these humors can be expelled by perspiration, then there is no *methodus medendi* comparable to the one which we have described as pursued at Graefenberg. The profuse sweating, induced in the manner which we have mentioned, is in every respect much preferable to what may be excited by severe bodily exercise, or by the internal use of any sudorific remedies. Then, too, the bold practice of immersing the patients while in a state of profuse perspiration into cold water must, if no evil consequences ensue from the sudden transition, most powerfully invigorate and harden the system.

Let it be remembered that copious libations of the pure element at the same time form a necessary part of the treatment. Dr. P. says that no patient should drink less than at least twelve tumblerfuls during the course of the day: some take as many as twenty-five or even thirty. In some obstinate cases, this practice has been continued for upwards of twelve months, and ultimately with complete success.

*Exercise,* also, is a necessary part of the regimen, as the vigour of the circulation cannot be kept up without it.

In conclusion, we shall briefly describe the regimen of a day at Graefenberg, as pursued by Dr. *Bigel* and other patients, under the directions of Dr. *Priessnitz.*

At four o'clock in the morning, a servant comes to roll you up in blankets. In Summer you will probably commence to sweat in from half an hour to an hour. After having sweated for about a couple of hours you go to the bath. On coming from the bath, you walk about, and drink occasionally a glassful or two of cold water, until the breakfast hour arrives. When this is over, you again walk about and drink more water. About eleven o'clock you go to the *douche* bath, and return at about noon, the hour of dinner. After this repast, the parties usually form themselves into different groupes either in the pleasure-grounds or elsewhere. About four o'clock is taken the last promenade, which may be called the sudorific one, as it consists in walking briskly up and down a hill, so as to encourage a very free perspiration. A cold bath is again taken; then a little exercise after the bath, and at eight o'clock supper is ready. A ball, a concert, or another promenade in the grounds closes the evening. Before retiring to bed, a hip-bath is used, the wetted compresses are placed on his bed, and the person then lays himself down to sleep.

For the first week or ten days, the patients are not subjected to so severe a regimen as afterwards, when their constitutions have been in some degree prepared, and are already considerably fortified. The sweating, for example, is not continued at first for more than an hour, and they do not remain above from five to fifteen minutes in the cold or in the douche bath.

Even with these precautions the regimen is in most cases found to be very exciting, and to induce a considerable febrile reaction in the system, and not unfrequently also diarrhœa, vomiting, and the development of abscesses, boils, and other forms of cutaneous eruption. The number of boils that make their appearance is sometimes surprising; from fifty to a hundred have been counted in one patient. They are considered as so many issues for the discharge of the morbid humors of the system through the cutaneous exhalants.

When the number or size of these abscesses is great, an abatement of the bathing regimen is generally necessary: the douche and the plunge baths are to be used for a shorter time, as these are powerful agents in promoting the derivation of the humors in the system towards the surface; but the sweating part of the regimen is continued as before, because thereby a portion of the vitiated humors, which would otherwise be discharged by the abscesses, is got rid of by the exhalants of the skin.

---

### Physiology of Ventriloquism.

Before explaining what seems to be the true interpretation of this curious *trick* of the voice, let us very briefly allude to the opinions which have been held on the subject by various writers....... "There can be very little doubt," says M. *Colombat,* "but that many of the oracular prophetesses, as those at Delphi, &c. were more or less adroit adepts in ventriloquism. So was the Witch of Endor, who evoked the ghost of Samuel at the bidding of Saul, and probably also the Pythoness mentioned in the Acts of the Apostles. As, in those times, whatever could not be explained was at once referred to the doing of the devil and his ministers, the ventriloquist was generally regarded as a person under the influence of demoniacal possession. It was believed that the voice came from the belly—hence, indeed, the origin of the word."

*Haller* and others attempted to explain ventriloquism by saying that the voice was formed during the act of inspiration.

In 1770, Baron *Mengen,* who was a ventriloquist himself, said that, in his opinion, the art consisted in keeping the tongue pressed firmly against the teeth, and in making them and the left cheek a circumscribed cavity, in which the voice was formed with the air held in reserve within the throat. The sounds had thus, he said, an obscure hollow tone, which gave the idea of their issuing from the belly. At the same time it is necessary to draw in the breath as seldom as possible, and to manage the expenditure of the air which has been inspired.

MM. *Dumas* and *Lanth* consider ventriloquism as a rumination of the voice, which, after being formed in the larynx, is thrown back into the chest, where it assumes a peculiar tone and acquires, on re-issuing, that dull and seeming-distant sound which gives rise to the illusion.

M. *Comte*, our (French) celebrated ventriloquist, tells us that, as far as he can judge, the voice is formed as usual in the larynx, but that the play of the other parts of the vocal apparatus modifies it, and that the act of inspiration directs it back into the thorax, where it resounds.

Sir *John Herschell*, in his Treatise on Sound, alludes to ventriloquism as being an art founded on the property of sound being not conveyed in a straight line, and on the difficulty therefore of the ear judging of the direction from which the sonorous undulations proceed. According to this distinguished philosopher, this incapacity of the ear, by which sounds which have a very simple and easily-explicable origin acquire a strange and unnatural character, depends not upon any imperfection of the auditory organs, but altogether upon the very nature of sound, whose "angle d'incertitude" is ever-varying according to the state of the atmospheric air and the nature of the surrounding objects. He adds that the voice of the ventriloquist is formed in the throat without the co-operation of the mouth and lips—the difference between the sounds so produced and those formed by the natural mechanism of the voice occasioning the deception.

The last opinion that we shall quote is that held by Dr. *Lespagnol*, and is the one which seems to us to be by far the most correct. He supposes that it is mainly by the aid of the velum palati preventing the escape of any of the air by the nasal passages that the ventriloquist is enabled so to modify the sounds of his voice as to produce the illusion. The only difference, says he, between the near and the distant-seeming voice is, that in the first we hear the sounds that come from the mouth and the nose, whereas in the second they proceed solely from the cavity of the cheeks. Indeed any one may, in a certain degree at least, verify the correctness of Dr. *Lespagnol's* opinion upon himself. No peculiar conformation of the respiratory or vocal organs is required; all that is necessary is that there should be a certain pliancy or mobility of the upper part especially of the vocal apparatus, and a sufficiently ample expansion of the lungs; and it will be found that with moderate practice almost any person will attain some skill in the tricks of ventriloquism. M. *Colombat* tells us that he has taught himself the art to a certain extent in the following manner. After filling the lungs completely by taking in a very deep breath, he contracts as powerfully as he can the velum palati, the pharynx, the base of the tongue, and all the muscles of expiration, in such a manner that the emission of the voice is effected by expelling as little as possible of air from the lungs, and at the same time so that the sounds produced do not re-echo in the nasal passages as in ordinary speaking, but in the cavity of the mouth only. This result is to be obtained with tolerable ease by forced contractions of the velum palati, and of all the muscles of the abdomen, chest, and neck. At the same time to render the voice as far seemingly-distant as possible, it will be found useful to lower its pitch and soften its tone, by raising the point of the tongue towards the uvula, in such a manner that the concave surface which the organ then presents may act as the *sourdine* (the little pipe in the mouth of a trumpet, &c. to lower the tone) of a wind instrument, or as the finger of the player with a stringed one.

According to this explanation of ventriloquism, the great secret of success depends upon preventing the air from escaping at all by the nose, and upon causing it to come entirely from the month in a slow and forced manner.

The voice so produced is dull, and has the feeble tone of a sound from a distance. To add to the delusion, if we wish to impart to the voice the tone of coming from any given spot, we have only to direct the attention of the listeners to that spot, and then to speak in that direction, elevating the velum palati in a greater or less degree, in order that the voice may at pleasure seem to approach or recede. It is always essential to success to keep the lips and jaw as much as possible motionless; and the ventriloquist, to prevent his audience from perceiving the slight movements of these parts, will do well to stand sideways to them, so that the profile only of his face can be seen. Lastly, every ventriloquist must be more or less of a good mimic, and have the talent of imitating various sounds with his natural voice.

It is well to add that in vain he might exercise all the resources of imitation and of artificial pronunciation, if sound were conveyed, like light, in straight lines, and if the ear could appreciate its direction as correctly as the eye can that of the luminous rays.

At the present time there is in France a ventriloquist, M. *Vattemare,* who is almost equal to the famous *Fitzjames,* whose extraordinary exhibitions of mimetic talent used to astonish the Parisians and Londoners at the beginning of the present century.

Those who wish to know more about the subject will do well to consult the register of the Academy of Sciences (for January 1771, p. 406), the work of the Abbé de la Chapelle, intitled *Le Ventriloque or L'Eregastrimythe,* and also the numbers of the *Revue Britannique* for December 1828, and March 1831.—*Gaz. des Medecins.*

---

### On the New (decimal) System of Weights in France.

Since the beginning of the present year, the French government has ordered that the old system of weights used by chemists and druggists, and consisting of grains, scruples, drachms and ounces be abolished, and be replaced with a decimal system as follows.

| | | |
|---|---|---|
| One grain | equivalent to | 5 centigrammes. |
| 10 grains | ,, | 50 ,, |
| 20 ,, | ,, | 1 gramme. |
| 30 ,, | ,, | 1 gramme and 50 centigrammes. |
| 40 ,, | ,, | 2 grammes. |
| 50 ,, | ,, | 2 grammes and 20 centigrammes. |
| One scruple (24 gr.) | | 1 gramme and 30 centigrammes. |
| One drachm | ,, | 4 grammes. |
| Two drachms | ,, | 8 ,, |
| Half an ounce | ,, | 15 ,, |
| One ounce | ,, | 30 ,, |

It will be observed that there is an exact analogy between this decimal system of weights and the existing division of money in France. Thus the *franc* consists of 20 sous or of 100 centimes, just as the *gramme* consists of twenty grains or of 100 centigrammes.

By bearing this simple rule in mind, the medical practitioner and druggist will at once be able to reduce the former weights to the present decimal standard. Thus, if he wishes to prescribe 12, 15 or 18 grains of a medicine, he has only to substitute the idea of sous for grains; and by reducing the sous into centimes he may reduce, by this simple operation, the grains of any medicine which he may wish into centigrammes.

Thus twelve grains make 60 centigrammes, 15 grains 75 centigrammes, 18 grains 90 centigrammes, 24 grains 1 gramme and 20 centigrammes; just as in 12 sous there are 60 centimes, in 15 sous 75 centimes, in 18 sous 90 centimes, and in 24 sous 1 franc and 20 centimes. Moreover, we have only to substitute the idea of grammes for that of francs, to discover at once the number of grammes which any number of grains compose. Thus 100 grains make five grammes, as 100 sous make five francs; 530 grains make 17 grammes and 50 centigrammes, as 530 sous make 17 francs and 50 centimes.

The decimal numeration is therefore by no means difficult to keep in mind, and by rejecting all other denominations but the two of *grammes* and *centigrammes* the memory is not at all perplexed. In place of using, as hitherto, the term *decagramme* to denote ten grammes, and then speaking of one, two, three decagrammes, it is far better to say at once 10, 20, and 30 grammes; and in the same way, instead of using the term *decigramme*, as has often been done, to denote 10 centigrammes, we have only to say 10 centigrammes at once, and so on, as 20, 30, 40, 50 centigrammes in place of one, two, three, four, and five decigrammes.

By this simple mode of numeration, we reduce the scale of weights to tally with the decimal scale of measures which has been established for a length of time, and is now perfectly understood by every one. The *metre* is, it is well known, the primary or standard measure of length; but its multiple *decametre* and its fraction *decimetre*—denoting the one 10 metres, and the other the 10th part of a metre—are now no longer used. For example, we do not talk of a man's height being one metre and seven decimetres, but one metre and seventy centimetres, nor of any thing costing one sous and 7 decimes, but one sous and 70 centimes.

A few words on the scruple, drachm, (*gros*), and ounce.

A *scruple* represents 24 grains: to reduce this to the present decimal standard we have only to remember the value of 24 sous; and as they make one franc and 20 centimes, so the scruple is equivalent to one gramme and 20 centigrammes.

The value of the drachm (72 grains) calculated on the bases of five centigrammes to one grain, would be three grammes and 60 centigrammes; but as its value is, according to the *poids de marc*, three grammes and 82 centigrammes, and, according to the Pharmacopœia, three grammes and 90 centigrammes, it has been determined to fix it now as an equivalent of an entire number, viz. four grammes or 80 grains. Nothing can therefore be more easy than to reduce drachms into grammes: we have only to multiply the number of the former by four: thus two drachms of any medicine are equivalent to eight grammes, three drachms to twelve grammes, and so forth.

The value of the ounce has been differently estimated. According to the *poids de marc*, it is equal to 30 grammes and 59 centigrammes; according to the Pharmacopœia to 32 grammes, and according to the metrical pound to 31 grammes and 25 centigrammes. At the suggestion of M. *Double*, in his luminous report submitted to the Academy of Medicine, it has now been fixed at the rate or value of 30 grammes exactly, excluding thus the additional 59 centigrammes, or about 11 grains of the *poids de marc* standard. To reduce the ounce into grammes is very simple: it need only be multiplied by 30, or if you prefer by 3, adding a cypher to the product. Thus four ounces of any medicine are equivalent to 120 grammes, six ounces to 180 grammes, and so forth.

Hitherto all has been abundantly simple; but a difficulty remains. This is to fix the value of the pound. If the ounce be 30 grammes, and 16 ounces be still considered as equivalent to one pound, the latter must be rated at 480 grammes; but, for various considerations which we cannot afford space at present to canvass, it has been judged better to rate it at 500 grammes; by which valuation it is rendered equivalent to 16 ounces and 20 grammes.—*Bulletin Generale de Therapeutique.*

### Professional Aphorisms.

1. The "savoir dire," the "savoir faire," an agreeable exterior and good manners, the knowledge of the world, a certain "je ne sais quoi," which pleases and attracts, are turned to excellent account by some physicians. But it will not do to examine such gentlemen too narrowly; we must not blow too strongly upon this froth; for the prestige quickly vanishes. These qualities are, indeed, to the character what embroidery is to a garment, whose web is of no great value.

2 "*An enlightened ignorance;*"—these words, although seemingly contradictory, express an important truth. It is given but to very few to reach this high degree of philosophic truth.

What study, what watchings, what meditation, how much judgment and modesty are required to know that we know but little, to estimate at their real value the acquisitions of science, to arrive at length at those limits where it is written, "*unknown.*" *Montaigne,* with much truth, distinguishes "the abecedarian ignorance and the doctoral ignorance;"—the latter requires a whole lifetime's labour to attain to.

3. *Bloodletting* is a very efficacious remedy in pneumonia; *tartrate of antimony* in large doses will often cure this disease; *opium* also may claim considerable success; and *musk* has saved not a few cases. But tell us, you say, with precision when are we to resort to bleeding, when to antimony, when to opium, and when to musk? We are almost compelled to admit that we cannot. It is the same with almost all pathological affections.

Shall we ever see the time, when we shall no longer be acting with hesitation and uncertainty, or pronouncing upon vague symptoms, and groping our way upon mere conjectures?

4. What is the cause of the bitterness of one physician against another? why does he blame him in every thing, and on every occasion? The truth is, he is occupied with the same subject, and he has been less successful. Do you not see the caterpillar abusing the work of the silkworm?—and yet the caterpillar can spin also.

Oh! my friends, guard against medical envy; it is a case of cancerous pathology, which eats its way deeper and deeper, until the whole system is contaminated.

5. The word *Finis* is, after much labour, placed at the end of your book; you have spared neither labour nor study to render it useful and interesting; it is learned and profound, well made and well written. But do you really imagine that you are at the end of your troubles? Remember that it must be printed; and here you will meet with much bother and perplexity. Then comes the publishing; another source of embarrassment and anxiety. At length, the book is published, advertised, &c. What does it want now? A thing rather rare in the present day—readers.

6. It is really absurd and ridiculous to see ourselves so often outstripped in the medical race by dolts and fools; and yet it is a disgrace and a reproach to succeed after the fashion of some people.

7. There are some writers, whose language, by being strong, compressed, and profound, exacts so much from the thought, that it is called obscure and unintelligible. The author of the *New Elements of the Science of Man* is an example of this style. A physician once said to this great man;—"your book is much too difficult to be understood." "Patience," replied *Barthez*, "I am preparing an edition which will be so clear that every ass will be able to drink from it."

8. The *genus irritabile* is found not only among poets and artists; a large proportion of medical men belong to the same category.

Nowhere is *amour-propre* more ticklish, more sensitive, more galvanic than among them: hence the endless disputes, the petty malice, and the secret love of spite and even of calumny, so prevalent amongst us.

We must manage and accommodate truth itself to these irritable vanities, as we do the light of the sun to very delicate eyes.

My dear brethren, be kind and charitable to each other. The public, not over benevolent at any time to you, only laughs at your squabbles, and quacks all the time reap a rich harvest. Remember that you are the apostles of humanity; be also the sages of time.

9. It is a noble thought and nobly expressed; *Pulchra sunt quæ videntur, pulchriora quæ sciuntur, sed longe pulcherrima quæ ignorantur.* How true of animal physiology, and, indeed, of every branch of physical science! At present we see but in a glass darkly. Will it ever be permitted to man, in his present state of existence, to penetrate the mysteries of Nature more deeply?

10. "I was dogmatic at twenty, an observer at thirty, an empiric at forty, and now at fifty I no longer have any system:"—

So said *Bordeu*: and he is quite right: sooner or later in science, as in life, we arrive at that wisdom which almost resembles the effect of disenchantment. But it is not given to all to reach in practice this high point of medical philosophy.

An acute sense, much knowledge, a superior reason, and a rare talent of disentangling truth from fiction and from mere probability, are necessary to enable us to form a just appreciation of theories and principles, and of their application to practice. Whoever has not acquired these qualities is condemned, like the crowd, to follow the standard of another, and to fall into either an irrational scepticism, or an empirical routine, which is too often dignified with the appellation of experience.—*Gazette Medicale.*

---

### On the Cause of Milky Blood.

The experiments of *Lecanu, Bertazzi, Christison,* and others, have most satisfactorily shewn that this abnormal condition of the blood depends upon the presence of an unusual quantity of fatty matter which is suspended in the serum. While in healthy blood the proportion of fatty matter does not exceed from one to two parts in 1,000, it has been found occasionally in cases of milky blood, as high as 30, 40, or even 100 parts.

It is a curious subject of pathological chemistry to explain how this increase of fatty matter in the serum is induced.

It has been stated by some writers that fatty matter may enter the torrent of the circulation in two different ways—either along with the chyle by the thoracic duct, or by the branches of the vena portæ. *Schultz* found that the blood of this vein contained double the quantity of fatty matter that the venous blood elsewhere did. As a large portion of it is no doubt expended in the secretion of the bile, it must follow that, whenever this secretion is interrupted, a larger proportion than usual will remain uneliminated from the system, and will consequently become diffused over the entire circulating mass.

But independently of this cause of an accumulation of fatty matter in the blood, the same condition of this fluid may very probably arise from a rapid absorption of the subcutaneous fat, or from any cause which may tend to impede its deposition in the cellular tissue.

By attending to these suggestions, we may be able to explain how this morbid alteration of the blood may be observed in diseases which have little or no analogy with each other, and to anticipate, in some measure, the description of cases in which it will be most likely to occur.—*Annales de la Societé de Med. de Gand.*

---

### On Involuntary Seminal Discharges.

The second volume of *Lallemand*'s valuable work has been recently published, and, as the distinguished Professor of Montpelier is an authority on whatever he treats of, we shall briefly glance at its contents.

In addition to the most frequent and well known causes of involuntary seminal discharges, M. *Lallemand* mentions the immoderate or protracted use of purgatives, narcotics, cantharides, camphor, ergot of rye, coffee, &c. The following are his remarks on the effects of *smoking*........

"If habit deadens the effect of tobacco on the system, the accumulation of its effects and the daily repetition of these must induce more durable changes on various organs. The disturbance of the digestion has been long observed in confirmed smokers; but that of the generative organs has not attracted the attention that the subject deserves.

I am convinced that this must be much more frequent than is generally believed, if I may judge by the torpor into which the organs of generation fall, as soon as the narcotic action of the tobacco is experienced, and by the habitual indifference evinced by confirmed smokers for the society of women. Attentive observation by the physicians of certain countries would probably lead them to detect the influence of these clouds of tobacco smoke on the production of involuntary seminal discharges, upon the dreamy and melancholic habits of many of their acquaintances, and upon the peculiar character of their relations with the other sex."

After having examined the external or extrinsic causes of involuntary discharges, our author treats of those which are attributable to the influence of other organs of the system, and to that of a congenital predisposition. Among the first, he mentions *irritation of the cerebellum*—(in accordance with the phrenological doctrine as to the seat of the amatory feelings);—and among the second, he enumerates congenital phimosis, the immoderate length of the prepuce, accompanied with a rudimentary state of the genital organs, a state of anemia, hypospadias, certain primitive arrangements of the ejaculatory canals, and, lastly, hereditary formation.

Having discussed these topics, M. *Lallemand* alludes to the history of that "self-tormenting sophist," Jean Jacques Rousseau, and with much probability accounts for many of the follies and vices of his strange character in a manner not very creditable to the *sage* of Geneva. He remarks,

"These observations have recalled to my memory many singular and bizarre traits which had often occurred to my mind in youth, while devouring the pages of this celebrated writer; they have also brought back the interminable and envenomed discussions between his admirers and his detractors in reference to his character, his opinions, and his actions. I have lately re-read these fascinating writings with a very different purpose; and I am now fully convinced that I was not mistaken as to the true cause of the solitary walks of *Rousseau*, of his ambulatory life, his savage misanthropy, and of his strange and paradoxical opinions on the civilization of mankind. We have only to read his Confessions to be assured of all this."—*Des pertes Seminales Involuntaires, par Dr. Lallemand.* 1839.

### IODURET OF IRON IN THE TREATMENT OF SYPHILITIC ULCERS.

M. *Baumes,* head surgeon of the hospital at Lyons, has of late used this new ferruginous preparation with most satisfactory results in the treatment of old and obstinate syphilitic ulcers, especially when the state of the system of the patient was at the same time feeble and scrofulous. He administered it in the form of pills with Thebaic extract, increasing the dose of the ioduret from two or three to twelve or twenty grains in the course of twenty-four hours. Along with the cicatrisation of the sores, the improvement of the general health was most remarkable; the appetite improved, the muscular strength increased, and the complexion acquired the florid hue of vigorous health. The salt, no doubt, is taken into the circulation, and acts on the blood itself, as well as on the capillary vessels in every part of the body.

### POISONING FROM ARSENIC SUCCESSFULLY TREATED WITH THE PEROXIDE OF IRON.

In a recent number of the German Journal *Medicinishe Annalen,* we observe a report of the successful administration of the hydrated peroxide of iron as an antidote to poisonous doses of arsenic. Five persons, three of whom were children, eat some soup in which powdered arsenic had been accidentally mixed. The symptoms were, as usual, excruciating tormina in the bowels, painful vomiting, great prostration, and excessive feebleness of the pulse, &c.

After the third dose of the peroxide had been taken, these symptoms were much mitigated, and under the use of demulcent and quieting drinks, all the patients ultimately recovered.

In another case alluded to by the reporter, a similar success, we are told, followed the exhibition of this ferruginous preparation.

Again, in a recent number of the French Journal *de Chimie Medicale,* M. *Batilliat* has reported the particulars of a case which occurred in his practice; but, as the quantity of the poison swallowed was in all probability very small, it seems unnecessary to give the particulars. The dried hydrated peroxide of iron was administered in doses of a spoonful at a time; and after the second or third dose the vomiting and other unpleasant symptoms ceased. M. B. recommends the following as a good method for obtaining the moist peroxide in a short space of time.

Let a quantity of iron filings be put along with diluted nitric acid into a large vessel, and heated over the fire; add some water to the solution and decant it off to separate the undissolved filings; then pour into it ammonia to excess, and again heat the liquor over the fire to get rid of the redundant alkali, and also for the purpose of giving a greater degree of cohesion to the precipitate. The clear liquor should then be drawn off by means of a syphon, and the peroxide should be washed repeatedly with hot water, which at first should be acidulated with vinegar: lastly, the precipitate is to be drained by being put upon a linen cloth.

In this way we can procure a sufficient quantity of a *bouillie* of the peroxide in the course of a couple of hours or so.—*Bulletin de Therapeutique.*

### German Treatment of Local Paralysis.

Several German writers very strongly recommend the use, internally as well as externally, of phosphorus in cases of paralysis of the optic and auditory nerves.

In a case related by M. *Dezeimeris* in his late elaborate memoir on diseases and injuries of the frontal sinuses, where deafness and blindness on the left side supervened upon a severe injury of the head, he resorted to this treatment with success.

He prescribed as follows :

℞. Phosphori gr. j. solve in

Olei animalis æther. ℥j.

Olei caryophyll. ℈j.

Dose.—Three drops, to be gradually increased to 20, to be taken on a piece of sugar night and morning. And

℞. Phosphori gr. ij.

Olei animalis æther. ℥j.

Olei cajeputi ℥ss. Solve.

The eyelids to be well rubbed with this embrocation three or four times daily.

A blister also was applied upon the mastoid process and kept open.

In four weeks the patient had quite recovered his hearing and sight.—*L'Experience.*

---

### Hepatic Abscess; Discharge of a Biliary Calculus.

Mad. N., 73 years of age, presented the external signs of an organic affection of the liver. The pain in the right hypochondrium became excessively severe, and high febrile symptoms came on.

After a few days, a phlegmonous swelling made its appearance at the seat of the pain ; this quickly assumed all the signs of an abscess, so as to induce the surgeon in attendance to make an opening into it. A large quantity of purulent matter mixed with bile and blood flowed out, and to prevent the healing of the wound, a tent was introduced and left in it. After the lapse of a few days, on introducing a probe along the fistula, a foreign body was felt distinctly. The wound being enlarged, this was extracted and proved to be a biliary calculus of nearly three inches and a half in diameter.—*L'Experience.*

---

### On Artificial Nipples of Ivory.

The following remarks are from a report of Messrs. *Dubois, Capuron* and *Villeneuve*—three of the most distinguished accoucheurs of Paris—on the artificial nipples recently invented by M. *Charriere,* an ingenious cutler of the metropolis.

"In the construction of these nipples, he uses ivory which has already been made soft and flexible by a process that has been long known : they are more solid and more durable than those hitherto employed. They are sufficiently resisting not to be flattened by the lips of the infant, and yet not too hard to fret and inconvenience them. They are easily kept clean by merely shaking them about in water, and with this simple precaution it will be found that they are not apt to communicate any unpleasant smell or taste to the milk. Besides, they can be readily attached to any sort of sucking dish or bottle, if the child is brought up by the hand and not suckled by the mother.

To preserve the flexibility of the prepared ivory, all that is necessary is to keep it from the contact of the air by placing the nipple under a glass, or by wrapping it round with a damp cloth. In fact we (the reporters) think highly of these new contrivances, and already, we are told, they are extensively used both in public establishments and in private practice."

(We have observed in some of the recent French journals, that the prepared flexible-made ivory has been manufactured into bougies for the male urethra; but whether they possess any peculiar advantages we do not know.

Excellent artificial nipples are now made with prepared caoutchouch : they are entirely destitute of any unpleasant smell or taste, and have quite a fleshy feel to the lips when sucked with.—*Rev.*)

---

### On the Preservation of Bodies for the Purposes of Dissection.

We are informed by Dr. *O'Shaugnessy* of Calcutta that since he has tried the practice of injecting bodies with a solution of arsenic—as recommended by Dr. *Tranchina* of Palermo, and Dr. *Dudley* of the U. S.—the study of anatomy has been pursued with greater ease on the banks of the Ganges than in London or Paris.

Dr. *Dujat,* of the *Ecole Pratique* in the latter metropolis, has for two years past adopted this plan, and reports very favourably of it, as affording by far the most efficient means of counteracting putrefaction. He says :—

.......... "The brain, after several weeks, was as firm as it usually is in a post-mortem examination ; and the pathological lesions of several organs retained all their accustomed appearances. The arm of a subject injected with the arsenical solution last March, (1839) was three months afterwards in a state of perfect preservation, and subsequently it was allowed to dry, the muscles retaining their usual dark red colour.

Even when putrefaction has commenced, the process will be arrested, and the decayed parts will recover much of their firmness and integrity, besides losing all their offensive odour. This process therefore affords a most convenient and effectual means of preserving pathological preparations when the weather is very hot or when we cannot put them up at the time. In preparing skeletons with their natural ligaments, as well also as the skins of animals, there is no antiseptic application so good as that of the arsenical solution."

Dr. *Dujat* seems not to have much fear that the employment of arsenic in this way is likely to prove at all poisonous to dissectors. "Indeed I am persuaded," says he, "that it renders the hands less liable to suffer from any wounds or punctures in dissection ; for the minute quantity of arsenic that could be absorbed in this way can frighten only the homœopathist ; and the experience of several anatomists in Paris, Calcutta, and at Levington in America, has shewn that there is little or no danger."

The strength of the solution used for injection by Dr. D. is considerably weaker than that recommended by *Tranchina* : it is 125 *grammes* of the arsenious acid to 1½ *kilogrammes* (or 1500 grammes) of water. It may be injected into either one of the crural or of the carotid arteries. For ordinary dissections, one injection will be found sufficient ; but when our object is to preserve a body for a great length of time, it should be repeated a second or even a third time.

The solution of arsenic is altogether preferable to that of corrosive sublimate, as this latter salt is apt to become decomposed, being converted into the protochloruret and ultimately into the metallic state.—*Journal de Chimie.*

